# Supplementary material for: Enhancing early detection of neurological and developmental disorders and provision of intervention in low-resource settings in Uttar Pradesh, India: study protocol of the G.A.N.E.S.H. programme
Source: BMJ Open. 2020 Nov 3;10(11):e037335. doi: 10.1136/bmjopen-2020-037335 (PMC7640505; doi:10.1136/bmjopen-2020-037335)
Supplement: Author's manuscript [file bmjopen-2020-037335.draft_revisions.pdf]

BMJ Open is committed to open peer review. As part of this commitment we make the peer review history of every article we publish publicly available.

When an article is published we post the peer reviewers' comments and the authors' responses online. We also post the versions of the paper that were used during peer review. These are the versions that the peer review comments apply to.

The versions of the paper that follow are the versions that were submitted during the peer review process. They are not the versions of record or the final published versions. They should not be cited or distributed as the published version of this manuscript.

BMJ Open is an open access journal and the full, final, typeset and author-corrected version of record of the manuscript is available on our site with no access controls, subscription charges or pay-per-view fees (http://bmjopen.bmj.com).

If you have any questions on BMJ Open's open peer review process please email info.bmjopen@bmj.com

BMJ Open

Enhancing early detection of neurodevelopmental disorders and provision of intervention in low-resource settings in Uttar Pradesh, India: The G.A.N.E.S.H. programme

Journal:	*BMJ Open*
Manuscript ID	bmjopen-2020-037335
Article Type:	Protocol
Date Submitted by the Author:	30-Jan-2020
Complete List of Authors:	Toldo, Moreno; Kiran Society for Rehabilitation and Education of Children with Disabilities<br>Varishthananda , Swami; Ramakrishna Mission Home of Service<br>Einspieler, Christa; Medical University of Graz, Division of Phoniatrics<br>Tripathi, Neeraj; Kiran Society for Rehabilitation and Education of Children with Disabilities<br>Singh, Anshu; Ramakrishna Mission Home of Service<br>Verma, Surendra; Kiran Society for Rehabilitation and Education of Children with Disabilities<br>Vishwakarma, Kanchan; Ramakrishna Mission Home of Service<br>Dwivedi, Agyeya; Kiran Society for Rehabilitation and Education of Children with Disabilities<br>Gupta, Ritika; Ramakrishna Mission Home of Service<br>Karn, Sanjay; Ramakrishna Mission Home of Service<br>Kerketta, Nirmal; Kiran Society for Rehabilitation and Education of Children with Disabilities<br>Narayan, Ram; Kiran Society for Rehabilitation and Education of Children with Disabilities<br>Nikam Singh, Karuna; Joints n Motion (JnM) Academy<br>Rani, Sumitra; Ramakrishna Mission Home of Service<br>Singh, Akanksha; Ramakrishna Mission Home of Service<br>Singh, Divyanshu; Kiran Society for Rehabilitation and Education of Children with Disabilities<br>Singh, Krishna; Ramakrishna Mission Home of Service<br>Singh, Navin; Kiran Society for Rehabilitation and Education of Children with Disabilities<br>Singh, Neeraj; Kiran Society for Rehabilitation and Education of Children with Disabilities<br>Singh, Rishi; Kiran Society for Rehabilitation and Education of Children with Disabilities<br>Singh, Shyam; Ramakrishna Mission Home of Service<br>Srivastava, Rakesh; Kiran Society for Rehabilitation and Education of Children with Disabilities<br>Srivastava, Sandeep; Kiran Society for Rehabilitation and Education of Children with Disabilities<br>Srivastava, Sanjeev; Ramakrishna Mission Home of Service<br>Yadav, Gopal; Kiran Society for Rehabilitation and Education of Children with Disabilities<br>Yadav, Preeti; Kiran Society for Rehabilitation and Education of Children

	with Disabilities<br>Yadav, Sheshnath; Ramakrishna Mission Home of Service<br>Yadav, Sujata; Ramakrishna Mission Home of Service<br>Marschik, Peter; University Medical Center Göttingen, Department of Child and Adolescent Psychiatry and Psychotherapy; Medical University of Graz, Division of Phoniatrics
Keywords:	Paediatric neurology < NEUROLOGY, Community child health < PAEDIATRICS, Developmental neurology & neurodisability < PAEDIATRICS, PREVENTIVE MEDICINE, PUBLIC HEALTH

SCHOLARONE™
Manuscripts

I, the Submitting Author has the right to grant and does grant on behalf of all authors of the Work (as defined in the below author licence), an exclusive licence and/or a non-exclusive licence for contributions from authors who are: i) UK Crown employees; ii) where BMJ has agreed a CC-BY licence shall apply, and/or iii) in accordance with the terms applicable for US Federal Government officers or employees acting as part of their official duties; on a worldwide, perpetual, irrevocable, royalty-free basis to BMJ Publishing Group Ltd ("BMJ") its licensees and where the relevant Journal is co-owned by BMJ to the co-owners of the Journal, to publish the Work in this journal and any other BMJ products and to exploit all rights, as set out in our licence.

The Submitting Author accepts and understands that any supply made under these terms is made by BMJ to the Submitting Author unless you are acting as an employee on behalf of your employer or a postgraduate student of an affiliated institution which is paying any applicable article publishing charge ("APC") for Open Access articles. Where the Submitting Author wishes to make the Work available on an Open Access basis (and intends to pay the relevant APC), the terms of reuse of such Open Access shall be governed by a Creative Commons licence – details of these licences and which Creative Commons licence will apply to this Work are set out in our licence referred to above.

Other than as permitted in any relevant BMJ Author's Self Archiving Policies, I confirm this Work has not been accepted for publication elsewhere, is not being considered for publication elsewhere and does not duplicate material already published. I confirm all authors consent to publication of this Work and authorise the granting of this licence.

For peer review only

Note from the Editors: Instructions for reviewers of study protocols

Since launching in 2011, BMJ Open has published study protocols for planned or ongoing research studies. If data collection is complete, we will not consider the manuscript.

Publishing study protocols enables researchers and funding bodies to stay up to date in their fields by providing exposure to research activity that may not otherwise be widely publicised. This can help prevent unnecessary duplication of work and will hopefully enable collaboration. Publishing protocols in full also makes available more information than is currently required by trial registries and increases transparency, making it easier for others (editors, reviewers and readers) to see and understand any deviations from the protocol that occur during the conduct of the study.

The scientific integrity and the credibility of the study data depend substantially on the study design and methodology, which is why the study protocol requires a thorough peer-review.

BMJ Open will consider for publication protocols for any study design, including observational studies and systematic reviews.

Some things to keep in mind when reviewing the study protocol:

- Protocol papers should report planned or ongoing studies. The dates of the study should be included in the manuscript.
- Unfortunately we are unable to customize the reviewer report form for study protocols. As such, some of the items (i.e., those pertaining to results) on the form should be scores as Not Applicable (N/A).
- While some baseline data can be presented, there should be no results or conclusions present in the study protocol.
- For studies that are ongoing, it is generally the case that very few changes can be made to the methodology. As such, requests for revisions are generally clarifications for the rationale or details relating to the methods. If there is a major flaw in the study that would prevent a sound interpretation of the data, we would expect the study protocol to be rejected.

For peer review only

Enhancing early detection of neurodevelopmental disorders and provision of intervention in low-resource settings in Uttar Pradesh, India: The G.A.N.E.S.H. programme

Moreno Toldo,[1a] Swami Varishthananda,[2a] Christa Einspieler,[3a*] Neeraj Tripathi,[1] Anshu Singh,[2] Surendra K. Verma,[1] Kanchan Vishwakarma,[2] Agyeya Dwivedi,[1] Ritika Gupta,[2] Sanjay Karn,[2] Nirmal Kerketta,[1] Ram Narayan,[1] Karuna Nikam Singh,[4] Sumitra Rani,[2] Akanksha Singh,[2] Divyanshu Singh,[1] Krishna P. Singh,[2] Navin Singh,[1] Neeraj Singh,[1] Rishi Singh,[1] Shyam P. Singh,[2] Rakesh Srivastava,[1] Sandeep Srivastava,[1] Sanjeev Srivastava,[2] Gopal Yadav,[1] Preeti Yadav,[1] Sheshnath Yadav,[2] Sujata Yadav,[2] Peter B. Marschik[3,5,6,7]

[a] These authors contributed equally to the present manuscript (shared first co-authorship)

***Correspondence to**: Dr. Christa Einspieler, Professor of Physiology
Research Unit interdisciplinary Developmental Neuroscience (iDN), Division of Phoniatrics, Medical University of Graz, Auenbruggerplatz 26, 8036 Graz, Austria
Phone: +43 316 385-0; E-mail: christa.einspieler@medunigraz.at

Authors' affiliations:

[1] Kiran Society for Rehabilitation and Education of Children with Disabilities, Varanasi, India
[2] Ramakrishna Mission Home of Service, Varanasi, India
[3] Research Unit iDN (interdisciplinary Developmental Neuroscience), Division of Phoniatrics, Medical University of Graz, Graz, Austria
[4] Joints n Motion (JnM) Academy, Mumbai, India
[5] Department of Child and Adolescent Psychiatry and Psychotherapy, University Medical Center Goettingen, Goettingen, Germany

Keywords: cerebral palsy, development, general movement assessment, low income setting, neurodevelopmental disorders, nutrition

Word count: 4,866

For peer review only

For peer review only - http://bmjopen.bmj.com/site/about/guidelines.xhtml

ABSTRACT

Introduction: Around 9% of India's children under 6 are diagnosed with neurodevelopmental disorders (NDDs). The Prechtl General Movement Assessment (GMA) has proved invaluable for contributing to identify children at risk for NDDs.

Methods: This 3-year prospective cohort study will comprise 2,000 infants born across four districts of Uttar Pradesh, India. Community health workers, certified for basic and advanced levels of GMA, video record and assess the infants' general movements (GMs) twice, i.e. within 2 months after birth and between 3 and 5 months after term. In case of abnormal GMs and/or reduced motor optimality scores, infants are further examined by a paediatrician and a neurologist. If necessary, early intervention strategies (treatment as usual) are introduced. At 12-24 months, the children are examined by a paediatrician, a neurologist (Hammersmith Infant Neurological Examination), and community health workers (Trivandrum Developmental Screening Chart). Outcomes are normal or categorised as NDD in case of developmental delay, cerebral palsy, epilepsy, visual impairment, hearing impairment, and a high-risk for autism spectrum disorder. Primary objective: to relate the GMA to the outcome at 12-24 months. Secondary objective: to relate the GMA, the outcome, and the association between these two with (a) access to and frequency of antenatal care, (b) maternal illness and/or micronutrient deficit during pregnancy, (c) safety of child birth, (d) gestational age, (e) birth weight, (f) neonatal complications, (g) the infant's medical condition and (h) nutritional status.

Ethics and dissemination: G.A.N.E.S.H. has received clearance from the Indian Government Chief Medical Officers of Varanasi and Mirzapur and was approved by the Ramakrishna Mission Home of Service in Varanasi. GMA is a worldwide used diagnostic tool, approved by the Ethics Committee of the Medical University of Graz, Austria (27-388 ex 14/15). Apart from peer reviewed publications, we are planning to deploy G.A.N.E.S.H. in other vulnerable settings.

For peer review only - http://bmjopen.bmj.com/site/about/guidelines.xhtml

Strengths and limitations of this study

- The strengths of G.A.N.E.S.H. include the application of a non-intrusive, highly reliable assessment tool (the Prechtl General Movement Assessment, GMA) to assist early identification of a high risk for cerebral palsy and other neurodevelopmental disorders in a low-income geographical cohort.
- Measurement biases may occur because blinding is not possible: it has taken the medical doctors and other staff members involved 15 years to win the vulnerable population's trust, which is the basis for conducting a study as outlined here.
- Higher-order impacts may be underestimated as a follow-up period of 24 months may be too short for interventions to take effect, or because certain NDDs cannot be diagnosed with absolute certainty at this age.
- A refusal rate of approximately 20% might contribute to underestimation of the true NDD burden.
- NDDs, and developmental delay in particular, may occur after 6 months (after weaning from breastfeeding) because of undernourishment, and might therefore not be detected by means of GMA during the first months of life.

For peer review only

INTRODUCTION

Background and problem context

India has the world's largest birth ratio (about 26 million per year) and is experiencing dramatic improvements in infant and child survival, although neonatal and infant mortality rates are still high at 30 and 41 per 1,000 live births, respectively.[1] With a population of more than 199 million (ca. 16% of the national population), Uttar Pradesh is the most populous state in India. The 2011 census[2] showed that 59 million people in Uttar Pradesh live below the poverty line. The state's maternal mortality rate is the second highest in India with 285 maternal deaths per 100,000 live births. The under-five mortality rate is 90 per 1,000 live births.[3] Lack of antenatal care, unsafe child births, undernourishment, and lack of access to health care infrastructure are among the main risk factors for maternal and child morbidity and mortality. Neurodevelopmental disorders

For peer review only - http://bmjopen.bmj.com/site/about/guidelines.xhtml

(NDDs) in particular compromise the attainment of full social and economic potential at family and community levels. Their rate of occurrence ranges from 2.9% to 18.7% in 2- to 9-year-old children across five Indian regions, and its all-site pooled estimate is 9.2% for children younger than 6 years.[4] In other words, almost one in eleven children has at least one of the following NDDs: intellectual disability, speech and language disorders, epilepsy, neuromotor impairments including cerebral palsy, autism, hearing and/or vision impairment.[4] Pooled estimates are unaffected by gender or rural/urban residence but increase by up to 3 percentage points when adjusted for national rates of low birth weight and/or stunting.[4] The overall pooled prevalence of cerebral palsy per 1,000 children in India is 2.95 (95% CI 2.03 to 3.88).[5]

Apart from low birth weight (<2500g) and preterm birth (< 37 weeks' gestation), non-institutional delivery, a history of perinatal asphyxia (delayed cry and difficult breathing at birth), neonatal illness requiring hospitalisation, postnatal brain infections, and stunting are significantly associated with NDDs, which include cerebral palsy.[4,5] Several of these risk factors could be averted through public health intervention. There is strong evidence that early intervention improves functional outcomes for infants with NDDs and is economically cost-effective as it reduces the rate and severity of later impairments.[6,7] Early identification of neurodevelopmental impairment is therefore vital for ensuring timely referral to the limited resources provided for impoverished children. However, low-income communities, which are most vulnerable to NDDs, often lack programmes for early identification and intervention.[8]

Government programmes such as India's Integrated Child Developmental Services provide supplementary food, health and nutrition education, health check-ups, growth monitoring and child-specific services like immunisation. But to this day only 20% to 30% of people from marginalised groups access these services.[9] Non-governmental organisations, and especially their community health workers, are indispensable for improving the access to early identification and intervention programmes since they work hand in hand with Accredited Social Health Activists (ASHAs), Auxiliary Nurse Midwives (ANMs) and Anganwadi Workers (AWWs), all of whom are part of India's public health care system. It is therefore of utmost importance that community health workers acquire relevant knowledge and skills to be able to recognise early markers for NDDs and refer infants in need to paediatric and neurological examinations. This goal has been

For peer review only - http://bmjopen.bmj.com/site/about/guidelines.xhtml

supported by the Bill & Melinda Gates Foundation with a Grand Challenges Explorations grant for the development of smartphone-based applications as assistive tools.[10]

About the organisations involved

The impact is assessed by two non-governmental organisations, Kiran Society (KS) and Ramakrishna Mission (RKM), in ten blocks across four districts in the south east of Uttar Pradesh, India.

Kiran Society (KS) is a non-profit organisation situated near Varanasi (also known as Benares) on the banks of the river Ganges in Uttar Pradesh. Since 1990, KS has sought to empower marginalised groups and children with disabilities through rehabilitation, education, vocational training, social integration, and advocacy (www.kiranvillage.org). KS offers institution- and community-based rehabilitation services providing neurological examinations and interventions, physio-, occupational-, speech/language- and psychotherapy as well as corrective orthopaedic aids and appliances. Based on the WHO's community-based rehabilitation matrix and on three schemes, KS' community-based rehabilitation unit currently provides services for 200,000 people in the districts of Varanasi and Mirzapur. One of these schemes is the **G**eneral Movement **A**ssessment in **N**eonates for **E**arly Identification and Intervention, **S**ocial Support and **H**ealth Awareness (https://kiranvillage.org/?page_id=3450): G.A.N.E.S.H. was launched in 2018.

Ramakrishna Mission (RKM) is a worldwide organisation founded in 1900. Its Varanasi branch, the RKM Home of Service, has a 230-bed hospital, nine indoor wards and 15 outpatient departments with various diagnostic units. Supported by the WHO, the European Commission, the British Medical Association and others, RKM Varanasi launched its Health Promotion Programme in 2000. Its main objectives are health education, primary health care services and training of community-based personnel in Varanasi and nearby districts. Its 15 actions and research projects include the following three, which are particularly relevant for G.A.N.E.S.H.: (a) child health care education (esp. multimedia presentations on feeding practices, hygiene and sanitation); (b) training community health workers and traditional birth attendants in promoting mother and child health; and (c) mobile mother and child health clinics along with family planning and immunisation interventions. Since 2009, RKM has provided antenatal care and

For peer review only - http://bmjopen.bmj.com/site/about/guidelines.xhtml

gynaecological services performed by mid-level women health workers in rural areas around Varanasi.

Both organisations' community health workers use mobile tools to pursue a broad range of health education goals including subjects such as vaccination, nutrition, hygiene and sanitation, but most importantly, they provide regular personal care. In addition, both organisations assist the most socio-economically disadvantaged families (especially pregnant women and lactating mothers, infants and young children) in terms of food supplements containing vitamins, iron and calcium.

Project overview and aims

The present prospective study is being carried out in a population-based cohort of underprivileged infants in four districts of Uttar Pradesh, India. We are assessing the motor repertoire of individuals aged 1 to 5 months and its association with (a) pre- and perinatal risk factors, and (b) the neurodevelopmental outcome in the second year of life. The study is conducted by the two non-governmental organisations portrayed above, which have long worked together and with governmental organisations in the target area. Disadvantaged families know that KS and RKM provide assistance in case of need.

The staff involved in both organisations received basic and advanced training in the Prechtl General Movement Assessment (GMA)[11], a diagnostic tool for early assessment of the integrity of the young nervous system. Training was provided by a licensed tutor of the GM Trust (C.E.), who also supervises the GMA and data evaluation during the project. Infants identified as high risk for NDDs are further examined by a paediatrician at RKM (S.V.) and a neurologist at KS (M.T.), who have worked together closely for more than 10 years. Another paediatrician is volunteering to assess infants and children in paediatric camps. All three of them are GMA-trained. KS has a special Medical Fund to guarantee that infants in need receive further diagnostic examination such as EEG, X-ray, MRI, or even genetic testing. If indicated, early physiotherapy is provided by KS staff, while both KS and RKM's teams provide basic health education and counseling.

For peer review only - http://bmjopen.bmj.com/site/about/guidelines.xhtml

The main objectives of the present 3-year project are to (a) train community health workers to identify infants at risk for NDDs early on; (b) monitor further assessments in infants identified as high-risk for NDDs; and (c) initiate early and targeted intervention procedures. Early identification is based on detailed assessments of the spontaneous motor behaviour[11] of infants born in eight Varanasi villages, 19 Mirzapur villages, one Sonbhadra cluster of villages and one Azamgarh cluster of villages. Our primary objective is to relate the quantity and quality of movement and postural patterns – which will be determined by means of the Prechtl GMA[11] at 1 to 5 months – to the outcome at 12 to 24 months, which will be assessed by paediatric and neurological examination (including the Hammersmith Infant Neurological Examination[12]) as well as developmental testing (Trivandrum Developmental Screening Charts[13]). Our secondary objective is to relate the results to a number of predefined confounders. Specific questions will be whether (i) movements and postures at 1 to 5 months, (ii) the 12- to 24-month-outcome, and (iii) the correlation between (i) and (ii) are associated with (a) the access to and frequency of antenatal care, (b) a maternal micronutrient deficit during pregnancy, (c) the safety of child birth, (d) a low birth weight (< 2500g) and undernourishment of the infant, and (e) a delayed cry at birth.

METHODS AND ANALYSES

Study design

Prospective observational cohort study.

Study setting and profile

In 2019, G.A.N.E.S.H. was implemented in 29 (cluster of) villages in ten blocks of four districts of Uttar Pradesh, where the burden of poverty and undernutrition is particularly high: the Varanasi district (roughly 3,700,000 inhabitants; 1,535 km^2; eight villages in the Kashi Vidyapeeth block, which is covered by KS); the Mirzapur district (roughly 2,500,000 inhabitants; 4,521 km^2; twelve villages in the Majhawan and Shikhar blocks, likewise covered by KS; seven villages or cluster of villages in the Chhanbey, Haliya, Jamalpur, Lalganj, Majhawan, Pahari and Patehara blocks, covered by RKM); the Sonbhadra district (roughly 1,800,000 inhabitants; 6,788 km^2; one cluster of villages in the Robertsganj block, covered by RKM), and

For peer review only - http://bmjopen.bmj.com/site/about/guidelines.xhtml

the Azamgarh district (roughly 4,600.000 inhabitants; 4,054 km^2; one cluster of villages in the Lalganj block, covered by RKM).

Eastern Uttar Pradesh (particularly the districts of Mirzapur and Sonbhadra) has one of the lowest health indicators of India's eight socioeconomically backward states, also referred to as Empowered Action Group (EAG) states. Poor health is characterised by lack of nutrition, chronic diseases and a high prevalence of infectious diseases resulting not only in high mortality and morbidity but also in an increase of poverty due to out-of-pocket expenses and lost earnings.[14] The main sources of livelihood are agriculture, carpet weaving and pottery, but youth unemployment is high. According to the latest census from 2011[2], the literacy rate then was between 64% in the Sonbhadra district and 75% in the Varanasi district, with a 15 to 20% lower literacy rate in women.

The target area is characterised by a lack of trained medical staff willing to work in rural areas and a lack of health care infrastructure. Most of the care is provided by the family. Families who seek treatment will often turn to non-allopathic providers such as practitioners of Indian traditional medicine and quacks (i.e. unqualified and unskilled health practitioners pretending to have medical skills).[14] The lack of neurodevelopmental health professionals, particularly in rural areas, places specialist neurodevelopmental care out of the reach of most people. The G.A.N.E.S.H. programme addresses this critical issue and seeks to provide healthcare to families in need.

Study population

The target population of G.A.N.E.S.H. comprises approximately 50,000 people. As the birth rate in the target area varies from 19.9 to 28.7 per 1,000,[2] we expect to see 1,000 to 1,500 newborns per year. We aim to assess a cohort of approximately 2,000 infants born in the first and second years of the study (2019-2020) and collect their outcome data during the second and third years of the study (2020-2021).

Recruitment and baseline data collection

In order to discuss the procedure and aims of the G.A.N.E.S.H programme, community health workers of KS and RKM arranged meetings with ASHAs and ANMs in each block. ANMs are

For peer review only - http://bmjopen.bmj.com/site/about/guidelines.xhtml

village-based female health workers establishing the first contact between the community and health services; in remote areas, ANMs also conduct home deliveries;[15] usually each ANM is supported by four to five ASHAs. ASHAs are local women trained as health educators and appointed by the Ministry of Health and Family Welfare of India. Their tasks include trying to persuade women to give birth in hospitals, taking children to immunisation clinics, encouraging family planning, treating basic illnesses and injuries with first aid, and improving village sanitation.[15]

Each community health worker involved in the project is linked with one ANM and will be informed by them about each live birth in her village. Every Wednesday and Saturday, the government offers free services in primary health centres in our target areas, where ANMs and ASHAs check the infants' weight and growth, promote and assist with breastfeeding and other nutritional aspects, and vaccinate. Community health workers linked to G.A.N.E.S.H. can also recruit mothers with newborn and young infants there.

The community health worker approaches the parent(s) within 4 weeks after delivery and explains the G.A.N.E.S.H. project in detail, including, for example, mutual commitments. He/she hands out leaflets with relevant information provided in words (Hindi) and pictures. Parents/caregivers are assured of confidentiality and anonymity in reports and publications generated as part of G.A.N.E.S.H. Participation is voluntary, and families are assured that refusing to participate will not affect their access to care. If parents agree to participate, their (written) consent is obtained. Illiterate parents are informed in the presence of a literate witness and consent with their finger-print.

For peer review only

After recruitment, a community health worker collects the following data (in an interview and, if available, from the medical records of delivery): demographical data including the parents' age; socioeconomic background and caste; maternal medical condition especially during pregnancy and delivery; maternal nutritional status; access to and frequency of antenatal care; site of delivery; complications during labour; the infant's gestational age at birth and birth weight; neonatal complications including a delayed cry at birth (which indicates asphyxia); the status of breastfeeding; the infant's medical condition since birth (including diarrhea, fever, etc.); and the infant's nutritional status (weight, length, head circumference) at the time of the GMAs.

For peer review only - http://bmjopen.bmj.com/site/about/guidelines.xhtml

The Prechtl General Movement Assessment (GMA)

The Prechtl GMA[11] is widely regarded as the best clinical tool to predict cerebral palsy in infants under 5 months[16] and has proved invaluable for contributing to identify children at risk for cognitive dysfunction,[17] intellectual disability,[18,19] speech and language disorders,[20] and autism spectrum disorders[21].

GMA is a diagnostic tool for an early assessment of the integrity of the young nervous system based on observation of young infants' spontaneous movements. It is entirely non-intrusive and extremely time-and cost-efficient. For a reliable assessment (kappa values ranging from 0.88 to 0.92)[11,17], a 2- to 5-minute video of the infant is recorded following a number of basic principles (infant lying in supine position, comfortably dressed, not crying, etc.).[11] Performed by trained assessors, the summary estimates for sensitivity and specificity of GMA are 0.98 (95% CI: 0.74–1.00) and 0.91 (95% CI: 0.83–0.93), respectively.[22] GMA is ideal for the neurological assessment of young infants, especially in low- and middle-income countries that struggle to provide expensive medical equipment for diagnoses.[23]

From birth to the end of the second month postterm age, normal general movements (GMs) include a fluctuating sequence of arm, neck, trunk and leg movements. They wax and wane with changing intensity, speed and range of motion. Their onset and ending is typically gradual and smooth. Changing of direction and rotations across the trunk and limb axes are fluent and elegant.[11] From 3 to 5 months, GMs appear as fidgety movements with a very small amplitude and moderate speed of shoulders, wrists, hips and ankles in all directions and of variable acceleration.[11] Most infants will develop normally if fidgety movements are present and normal, especially if they co-occur with other smooth and fluent age-specific movement patterns.[11,17,24]

The neural mechanisms of GMs are specific neural networks, central pattern generators (CPG), which are most probably located in the brain stem. We believe that the CPGs for GMs are constantly modulated by more rostral parts of the brain. The resulting variable movements in turn modulate the CPG-activity by variable sensory feedback.[11,17] If a structural impairment of the young nervous system becomes functional, supra-spinal projections are impaired and reduce the modulation of CPG activity, resulting in less variable GMs.[24] Before 3 months of age, abnormal GMs are described as: (i) 'poor repertoire' (the sequence of movements and the intensity, speed and range of motion lack variability) or (ii) 'cramped-synchronised' (the limb and trunk muscles appear rigid and contract almost simultaneously, then relax almost simultaneously). As a sign of functional impairment, fidgety movements can be (iii) 'abnormal' (exaggerated in amplitude and speed), (iv) sporadically present (not exceeding 3 seconds[25]) or (v) missing

For peer review only - http://bmjopen.bmj.com/site/about/guidelines.xhtml

altogether at a post-term age of 9 to 16 weeks; (iv) and (v) are summarised as 'absent fidgety movements'.[11,24,25] Summary estimates of 0.98 for sensitivity and 0.91 for specificity make cramped-synchronised GMs followed by absent fidgety movements very reliable early markers of spastic cerebral palsy.[16,24]

The motor repertoire of infants aged 3 to 5 months consists of fidgety movements as well as other movement patterns such as swiping, wiggling-oscillating limb movements, kicking, arm and leg movements to the midline, fiddling, leg lifts, or arching.[26,27] The detailed GMA for 3- to 5-month-old infants comprises the following five subcategories: (i) temporal organisation and quality of fidgety movements; (ii) quality of other observed movement patterns; (iii) age-adequate movement repertoire; (iv) postural patterns; and (v) movement character. Adding the scores of the five subcategories reveals the motor optimality score (MOS)[11,27] with a maximum of 28 (i.e. best possible performance) and a minimum of 5. An MOS ranging from 25 to 28 is considered to be optimal; scores from 20 to 24 are mildly reduced; and an MOS below 20 requires intervention. A score below 9 indicates a very high risk for NDDs, especially for non-ambulatory cerebral palsy.[11,27] Various studies revealed that a reduced MOS was associated with later motor,[27-30] cognitive,[29,31] and language[20,29] impairments. A recent worldwide study on 468 children later diagnosed with cerebral palsy shows that such a detailed assessment of early movements and postures provides insight into a child's later functional capabilities and impairment.[27] Intra-observer reliability is high with intra-class correlation coefficients ranging from 0.80 to 0.98[27,28] and Cohen Kappa ranging from 0.87 to 0.91[30].

Training KS and RKM staff to use GMA

In February and November 2018, KS and RKM staff involved in G.A.N.E.S.H. (medical doctors, physiotherapists, medical social workers, community-based rehabilitation field workers, special educators, and one psychologist) received training from C.E.. They are now GM Trust-certified (basic and advanced level; www.general-movements-trust.info) to perform GMA. As a side note: for better readability we have summarised the professions of medical social workers, community-based rehabilitation field workers and special educators under the term 'community health workers' throughout the manuscript.

Performing GMA

For peer review only - http://bmjopen.bmj.com/site/about/guidelines.xhtml

After parental consent has been received, a GMA-trained KS/RKM staff member videotapes the infant's GMs according to GMA standards (the infant shows 2 to 5 minutes of active wakefulness, must not be crying, fussy or sucking on a pacifier, and must be lying in supine position without manipulation).[11] Video-recordings are preferably at two points in time: (a) during the first 2 months of life; and (b) during the fidgety movement period between the end of the 2nd month and the end of the 5th month postterm age (corrected for preterm delivery). Infants are videotaped in the parents' home, in health camps, or in a primary health centre. The initial evaluation is performed by the GMA-certified staff member who has recorded the video, the second evaluation is performed by the first author (M.T.), and a third evaluation by C.E., who isn't familiar with the medical history of the infant. C.E. supervises the GMA as licensed tutor of the GM Trust. In a pilot sampling approach, we recorded and assessed 400 infants revealing intra-class correlation coefficients ranging from 0.85 to 0.92 between the primary and secondary assessors and the supervisor.

Intervention programmes

The infant is referred to a targeted intervention programme if at least one of the following criteria is observed: (a) cramped-synchronised GMs during the first two months; in case of abnormal poor repertoire GMs during the first two months, GMA is repeated at 3 to 5 months; (b) absence of fidgety movements or exaggerated, abnormal fidgety movements at 3 to 5 months; (c) MOS <20; (d) asymmetries of any kind, especially asymmetric segmental wrist and/or finger movements[27]; (d) body weight and/or head circumference at or below the third age-related percentile on the WHO charts; (e) facial dysmorphism and/or malformations such as congenital talipes equinovarus, cleft palate; (f) acute illness; (g) any other parental concern.

Infants identified as high risk for NDDs and infants who need medical examination are referred to a paediatrician at RKM (S.V.) and/or a neurologist at KS (M.T.), who have worked together closely for more than 10 years. A second paediatrician is volunteering to assess infants and children in paediatric camps. All three of them are GMA-trained. KS has a special Medical Fund to guarantee that infants in need receive further diagnostic examination such as EEG, X-ray, MRI, or even genetic testing. If indicated, physio-, occupational-, speech/language therapists and special educators treat infants individually at home or at the Parents and Child Care Unit of KS.

For peer review only - http://bmjopen.bmj.com/site/about/guidelines.xhtml

Since G.A.N.E.S.H. has been launched, every staff member involved in interventions has received regular workshops and practical demonstrations of early intervention techniques.

Trained RKM and KS personnel also offers education and counselling on basic health, nutrition and hygiene. Severely anaemic or malnourished mothers and underweight infants are given supplementary nutritious food, vitamins and micronutrients (especially calcium and vitamin D, iron and folic acid tablets) as well as medical care. Infants who need intensive medical treatment are referred to the Ramakrishna Hospital or the district hospitals of Varanasi and Mirzapur. Families in need are invited early on to attend paediatric or rehabilitation camps providing free services, examinations and, if necessary, additional medication.

Output measures

We expect G.A.N.E.S.H. to improve health outcomes in children living in the target area. This goal requires stepwise procedures (Table 1).

In addition, we aim to answer the following research questions:

Primary research objective

Our primary objective is to relate the quantity and quality of movement and postural patterns assessed by means of the Prechtl GMA[11] (at 1 to 5 months) and MOS[27] (at 3 to 5 months) to the outcome at 12 to 24 months, which is assessed in paediatric and neurological examinations as well as developmental tests. The neurological assessment follows the protocol of the Hammersmith Infant Neurological Examination (HINE).[12] This method is easily performed and was designed for examining children between 2 and 24 months of age. It includes 26 items that assess cranial nerves, posture, movements, tone, and reflexes. Sensitivity and specificity are around 0.90.[32] The Trivandrum Developmental Screening Chart (TDSC)[13] is based on 17 test items from the Bayley Scales of Infant Development (Baroda Norms). It was validated both at hospital and community levels and has a sensitivity of 0.85 and a specificity of 0.91, which makes it a simple, reliable screening tool that can also be applied by community health workers.[33] In addition, two psychologists (one at KS, one at RKM) were trained to identify socio-

For peer review only - http://bmjopen.bmj.com/site/about/guidelines.xhtml

communicative deficits which, along with repetitive and restricted behaviours, may indicate an increased likelihood of developing autism spectrum disorder.

The outcome is either normal or indicates developmental delay, cerebral palsy, epilepsy, visual impairment, hearing impairment, or a high risk for autism spectrum disorder. In addition, each child's nutritional status is assessed and categorised as normal, underweight, wasted (weight-for-age Z-scores that are more than 2 standard deviations below the median of the WHO child growth reference standards) or stunted (with height-for-age Z-scores that are more than 2 standard deviations below the median of the WHO child growth reference standards). Head circumference Z-scores will also be documented.

Secondary research objective

Our secondary objective is to relate (i) age-specific movements and postures assessed at 1 to 5 months by means of GMA[11] and MOS[27], (ii) the 12- to 24-month-outcome assessed by means of HINE[12] and Trivandrum Developmental Screening Charts[13], and (iii) the correlation between (i) and (ii) to the following predefined confounders: (a) access to and frequency of antenatal care; (b) maternal illness and/or micronutrient deficit during pregnancy; (c) safety of child birth, complications during and after delivery, caesarean section and its cause; (d) gestational age (if available), otherwise preterm vs. full term birth; (e) birth weight; (f) neonatal complications with a focus on a delayed cry at birth (which indicates asphyxia); (g) infant's medical condition since birth (including diarrhea, fever, etc.) and particularly at the time of video recording; and (h) the infant's nutritional status (weight, length, head circumference as Z-scores) at the time of video recording for GMA.

Statistical analysis

First, we evaluate descriptive parameters (medians and interquartile ranges or percentages) to summarise sample characteristics. The MOS (and its subcategories) and the HINE are ordinal data, whereas the number of normal or atypical movements or postural patterns, the gestational age at birth, birth weight, assessment age, weight, length, head circumference Z-scores, weight-for-age Z-scores, weight-for-length Z-scores are interval data. All other pre- and perinatal data, GMA data and outcome data are analysed as categorical variables. Pearson's chi-squared test or

For peer review only - http://bmjopen.bmj.com/site/about/guidelines.xhtml

Fisher's exact test (two-sided) are used for comparisons between categorical variables. Odds ratios and 95% confidence intervals are calculated for (a) pre- and perinatal data that reveal a significant difference between normal and abnormal GMs, and (b) normal and abnormal GMs that reveal a significant difference between the categorical outcome variables assessed at 12 to 24 months. To determine whether two or more independent samples had the same distribution in MOS and/or HINE, we apply the Mann-Whitney *U* or Kruskal Wallis tests as we expect the data to be not normally distributed. We use the Wilcoxon signed-rank test to determine whether the quantities of normal and atypical movements and postures (dependent samples; assumed to be not normally distributed) were equally distributed. We use the Spearman rank-order correlation to determine the association between the MOS (at 3 to 5 months) and the HINE (at 12 to 24 months). All values of $p<0.05$ will be regarded as statistically significant. Data analyses are performed using IBM SPSS Statistics in its updated version.

Longer-term outcomes

The listed longer-term outcomes are those we expect to achieve in a follow-up evaluation period of 3 to 5 years, depending on the availability of funds. Details will be determined later, as they can change according to requirements. Long-term outcomes should reflect an improved child health and nutritional status, improved motor, cognitive, and language abilities, a better supply of vision and hearing aids and orthoses, proper management in case of epilepsy, access to MRI, EEG, X-ray and other diagnostic procedures, as well as adequate psychological treatment and counselling – in other words: a proper management of NDDs and a strengthened health system.

Patient and Public Involvement

It has not been appropriate to involve infants and their families or the public in the design, or conduct, or reporting, or dissemination plans of G.A.N.E.S.H.

Ethics and dissemination

After parents/caregivers have received detailed information on G.A.N.E.S.H., they are assured of confidentiality and anonymity in reports and publications generated as part of G.A.N.E.S.H. Participation is voluntary, and families are assured that refusing to participate will not affect their access to care. If parents agree to participate, their (written) consent is obtained. Illiterate parents are informed in the presence of a literate witness and consent with their fingerprint. All study

For peer review only - http://bmjopen.bmj.com/site/about/guidelines.xhtml

For peer review only

data are stored in secure databases. Paper files are kept in locked filing cabinets, and computer and video files are stored on password-protected servers. Only staff members associated with the G.A.N.E.S.H. programme have access to the data.

Results are submitted to peer-reviewed journals and presented at national and international conferences. Beyond that, we aim for G.A.N.E.S.H. to serve as a model for early identification of and intervention in children with NDDs or at risk for NDDs, to have a sustainably impact on health care plans for low-income settings. Therefore, dissemination to health care professionals and policy makers is crucial.

All results presented are group data; individual participants are not identifiable. We intend to analyse and publish GMA and MOS results as well as 12-to 24-month outcomes as stand-alone research articles in peer-reviewed journals. In accordance with our main aim to improve the quality and quantity of interactions with beneficiary families, the main research questions to answer and discuss, are:

1. Is it feasible for trained community health workers to launch G.A.N.E.S.H. and apply GMA in a rural, low-income setting? Key aspects: s*uccess of training, acceptance by ASHAs, ANWs, acceptance in the community, challenges, inter-scorer agreement, frequent staff meetings, expert supervision.* Limitations/risks: *parental refusal, cultural setting in case of male community health worker, migration of trained staff, priority of nutritional aspects over GMA.*
2. Does G.A.N.E.S.H. improve the frequency of home visits for infants and their mothers and the extent and level of counselling for families in need, and does it help to identify abnormal motor behaviour and malnutrition? Key aspects: *identification of abnormal GMs and reduced MOS, parental compliance with further paediatric and neurological examination, launching of targeted family-oriented intervention, targeted health education, provision of food supplementation and micronutrients.* Limitations/risks: *no compliance with further assessment and intervention because early markers are subtle; families see the need to consult a doctor only in case of (frequent) seizures, acute infection, or malformations like cleft palate and congenital talipes equinovarus. The need*

For peer review only - http://bmjopen.bmj.com/site/about/guidelines.xhtml

for prevention and early intervention is difficult to grasp for parents, and is often impeded by cultural hierarchical family structure.

3. Are we able to identify early on (especially at 3 to 5 months) children who will have an adverse outcome around 12 to 24 months of age? Key aspects: *predictive value of GMA, sustainable family acceptance and compliance with targeted strategies of early intervention.* Limitations/risks: *the nutritional status, especially after weaning from breastfeeding (second half of the first year of life) might have a greater impact on the development than the integrity of the nervous system determined by GMA.*

For peer review only

Acknowledgements: The authors are grateful to Professor Akmer Mutlu, Hacettepe University Ankara, Turkey for providing teaching material to the physiotherapy unit at Kiran Society; to Mrs. Sikha Sharma for basic insights into the principles of early intervention; to Mr. Amarendra Kumar and Mr. Vijay Pal for their support in community-based rehabilitation; to Dr Ruchi Pandey for her assistance in paediatric camps; to Mrs. Sangeeta J.K., founder and president of Kiran Society, for introducing the community-based rehabilitation approach among Kiran Society's activities and her ongoing support and inspiration; and to Miha Tavcar (www.scriptophil.at) for copy-editing the article.

Contributors: MT, SV and CE conceptualised G.A.N.E.S.H. and conceived the research questions. MT, SV, CE and PBM secured funding for the project and prepared the study protocol with feedback from all collaborators. MT, SVP and CE are principle investigators, PBM is co-investigator. NT and AnS supervise the data collection under the guidance of investigators. SKV, AnS, KV, AkS, and CE developed the data base for the project. MT, SV, NT, AnS, KNS, DS, NaS, and RaS supervise interventions applied in G.A.N.E.S.H. All authors read and approved the final protocol.

Funding: This work is supported by Bill & Melinda Gates Foundation (opp1128871), the Austrian Science Fund FWF (P25241, TCS24, KLI811), the GM Trust (WHEP23-8-5-16), the

Australian Cerebral Palsy Alliance (SEG00419), the Old Possum's Practical Trust, the Medical Fund of Kiran Society and the Ramakrishna Mission.

Competing interests:

Ethics Approval: G.A.N.E.S.H. has received clearance from the Indian Government Chief Medical Officers of Varanasi and Mirzapur and was approved by the Ramakrishna Mission Home of Service in Varanasi. GMA is a worldwide used diagnostic tool, approved for its retrospective and prospective application by the Ethics Committee of the Medical University of Graz, Austria (27-388 ex 14/15).

Provenance and peer review: Not commissioned; externally peer-reviewed.

Data sharing statement: This being a protocol no unpublished data are available.

REFERENCES

1. International Institute for Population Sciences (IIPS) and ICF. National Family Health Survey (NFHS-4), 2015-2016: India. Mumbai: IIPS, 2017. http://rchiips.org/NFHS/NFHS-4Reports/India.pdf (accessed Nov 25, 2019).
2. Census of India 2011. www.censusindia.gov.in (accessed Oct 3, 2019).
3. Annual Health Survey Report, Part I. Office of the Registrar General & Census Commissioner. Ministry of Home Affairs, Government of India, 2013.
4. Arora NK, Nair MKC, Gulati S, *et al.* Neurodevelopmental disorders in children aged 2–9 years: Population-based burden estimates across five regions in India. *PLoS Med* 2018;15:e1002615.
5. Chauhan A, Singh M, Jaiswal N, *et al*. Prevalence of cerebral palsy in Indian children: a systematic review and meta-analysis. *Indian J Pediatr* 2019;doi 10.1007/s12098-019-03024-0.
6. Spittle A, Orton J, Anderson P, *et al*. Early developmental intervention programmes post-hospital discharge to prevent motor and cognitive impairments in preterm infants. *Cochrane Database Syst Rev* 2015;11:CD005495.

For peer review only - http://bmjopen.bmj.com/site/about/guidelines.xhtml

7. Benfer KA, Novak I, Morgan C, *et al*. Community-based parent-delivered early detection and intervention programme for infants at high risk of cerebral palsy in a low-resource country (Learning through Everyday Activities with Parents (LEAP-CP): protocol for a randomized controlled trial. *BMJ Open* 2018;8:e021186.

8. UN World Health Organization (WHO). 58th World Health Assembly: Resolutions and Decisions, Annex. Geneva: World Health Organization 2005. Document WHA58/2005/REC/1. http://apps.who.int/gb/ebwha/pdf_files/WHA58-REC1/english/A58_2005_REC1-en.pdf (accessed at July 15, 2019).

9. Chakrabarti S, Raghunathan K, Alderman H, *et al*. India's Integrated Child Development Services programme; equity and extent of coverage in 2006 and 2016. *Bull World Health Organ* 2019;97:270-282.

10. Marschik PB, Pokorny FB, Peharz R, *et al*. A novel way to measure and predict development: a heuristic approach to facilitate the early detection of neurodevelopmental disorders. *Curr Neurol Neurosci Rep* 2017;17:43. doi 10.1007/s11910-017-0748-8

11. Einspieler C, Prechtl HFR, Bos AF, *et al*. *Prechtl's Method on the Qualitative Assessment of General Movements in Preterm, Term and Young Infants*. London, UK: Mac Keith Press 2004.

12. Haataja L, Mercuri E, Regev R, *et al*. Optimality score for the neurologic examination of the infant at 12 and 18 months of age. *J Pediatr* 1999;135:153-161.

13. Nair MK, George B, Philip E, *et al*. Trivandrum developmental screening chart. *Indian Pediatr* 1991;28:869-872.

14. Arokiasamy P, Gautam A. Neonatal mortality in the empowered action group states of India: trends and determinants. *J Biosoc Sci* 2008;40:183-201.

15. Nimmagadda S, Gopalakrishnan L, Avula R, *et al*. Effects of an mHealth intervention for community health workers on maternal and child nutrition and health services delivery in India: protocol for a quasi-experimental mixed-methods evaluation. *BMJ Open* 2019;9:e025774.

16. Novak I, Morgan C, Adde, L, *et al.* Early, accurate diagnosis and early intervention in cerebral palsy: advances in diagnosis and treatment. *JAMA Pediatr* 2017;171:897-907.

For peer review only - http://bmjopen.bmj.com/site/about/guidelines.xhtml

17. Einspieler C, Bos AF, Libertus ME, *et al*. The general movement assessment helps us to identify preterm infants at risk for cognitive dysfunction. *Front Psychol* 2016;7:406. doi 10.3389/fpsyg.2016.00406.

18. Herrero D, Einspieler C, Panvequio Aizawa CY, *et al*. The motor repertoire in 3- to 5-month old infants with Down syndrome. *Res Dev Disabil* 2017;67:1-8.

19. Einspieler C, Marschik PB. Regression in Rett syndrome: Developmental pathways to its onset. *Neurosci Biobehav Rev* 2019,98:320-332.

20. Salavati S, Einspieler C, Vagelli G, *et al*. The association between the early motor repertoire and language development in term children born after normal pregnancy. *Early Hum Dev* 2017;111:30-35.

21. Einspieler C, Sigafoos J, Bartl-Pokorny KD, *et al.* Highlighting the first 5 months of life: general movements in infants later diagnosed with autism spectrum disorder or Rett syndrome. *Res Autism Spectr Dis* 2014;8:286-291.

22. Bosanquet M, Copeland L, Ware R, *et al*. A systematic review of tests to predict cerebral palsy in young children. *Dev Med Child Neurol* 2013;55:418-426.

23. Tomantschger I, Herrero D, Einspieler C, *et al*. The general movement assessment in non-European low- and middle-income countries. *Rev Saude Publica* 2018;52:6. doi: 10.11606/S1518-8787.2018052000332.

24. Prechtl HFR, Einspieler C, Cioni G, *et al.* An early marker for neurological deficits after perinatal brain lesions. *Lancet* 1997;349:1361-1363.

25. Einspieler C, Yang H, Bartl-Pokorny KD, *et al.* Are sporadic fidgety movements as clinically relevant as is their absence? *Early Hum Dev* 2015;91:247-252.

26. Einspieler C, Marschik PB, Prechtl HFR. Human motor behaviour. Prenatal origin and early postnatal development. *J Psychol* 2008;216:148-154.

27. Einspieler C, Bos AF, Krieber-Tomantschger M, *et al*. Cerebral palsy: early markers of clinical phenotype and functional outcome. *J Clin Med* 2019;8:1616. doi: 10.3390/jcm8101616.

28. Zang FF, Yang H, Han Q, *et al*. Very low birth weight infants in China: the predictive value of the motor repertoire at 3 to 5 months for the motor performance at 12 months. *Early Hum Dev* 2016;100:27-32.

For peer review only - http://bmjopen.bmj.com/site/about/guidelines.xhtml

29. Einspieler C, Utsch F, Brasil P, *et al*. Association of infants exposed to prenatal Zika virus infection with their clinical, neurologic, and developmental status evaluated via the general movement assessment tool. *JAMA Netw Open* 2019a; e187235.

30. Bruggink JL, Einspieler C, Butcher PR, *et al*. Quantitative aspects of the early motor repertoire in preterm infants: do they predict minor neurological dysfunction at school age? *Early Hum Dev* 2009;85:25-36.

31. Butcher PR, van Braeckel K, Bouma A, *et al*. The quality of preterm infants' spontaneous movements: an early indicator of intelligence and behaviour at school age. *J Child Psychol Psychiatry* 2009;50:920-30.

32. Romeo DM, Ricci D, Brogna C, *et al*. Use of the Hammersmith Infant Neurological Examination in infants with cerebral palsy: a critical review of the literature. *Dev Med Child Neurol* 2016;58:240-245.

33. Nair MK, Nair GS, George B, *et al*. Development and validation of Trivandrum Developmental Screening Chart for children aged 0-6 years [TDSC (0-6)]. *Indian J Pediatr* 2913;80:S248-S255.

For peer review only

Table 1: Main procedures and outcome measures

Procedures	Output	
Training community health workers to identify infants at risk for NDDs early on	(1)	staff trained and certified in basic and advanced GMA
	(2)	agreement (a) between the scorers, (b) between the scorers and a GMA-trained neurologist, and (c) between the scorers and a GMA expert
Monitoring further assessments of infants identified as high-risk for NDDs	(3)	number of infants screened
	(4)	number of infants identified as high-risk for NDDs (abnormal age-specific GMs, reduced MOS)
	(5)	number of families supported
	(6)	number of underweight infants
	(7)	number of infants examined by a paediatrician and/or a neurologist; examination results
	(8)	number of (breastfeeding) mothers and infants who received micronutrient supply
	(9)	number of infants who received (financially supported) access to MRI, EEG, X-ray, and laboratory testing
Launching targeted intervention procedures early on.	(10)	number of staff trained in early parent oriented intervention
	(11)	number of infants referred to early intervention
	(12)	number of families referred to further parent training (around the infant's age of 6 to 7 months)

For peer review only

For peer review only - http://bmjopen.bmj.com/site/about/guidelines.xhtml

BMJ Open

Enhancing early detection of neurological and developmental disorders and provision of intervention in low-resource settings in Uttar Pradesh, India: Study protocol of the G.A.N.E.S.H. programme

Journal:	*BMJ Open*
Manuscript ID	bmjopen-2020-037335.R1
Article Type:	Protocol
Date Submitted by the Author:	28-Jul-2020
Complete List of Authors:	Toldo, Moreno; Kiran Society for Rehabilitation and Education of Children with Disabilities<br>Varishthananda , Swami; Ramakrishna Mission Home of Service<br>Einspieler, Christa; Medical University of Graz, Division of Phoniatrics<br>Tripathi, Neeraj; Kiran Society for Rehabilitation and Education of Children with Disabilities<br>Singh, Anshu; Ramakrishna Mission Home of Service<br>Verma, Surendra; Kiran Society for Rehabilitation and Education of Children with Disabilities<br>Vishwakarma, Kanchan; Ramakrishna Mission Home of Service<br>Zhang, Dajie; University Medical Center Göttingen, Department of Child and Adolescent Psychiatry and Psychotherapy; Medical University of Graz, Division of Phoniatrics<br>Dwivedi, Agyeya; Kiran Society for Rehabilitation and Education of Children with Disabilities<br>Gupta, Ritika; Ramakrishna Mission Home of Service<br>Karn, Sanjay; Ramakrishna Mission Home of Service<br>Kerketta, Nirmal; Kiran Society for Rehabilitation and Education of Children with Disabilities<br>Narayan, Ram; Kiran Society for Rehabilitation and Education of Children with Disabilities<br>Nikam Singh, Karuna; Joints n Motion (JnM) Academy<br>Rani, Sumitra; Ramakrishna Mission Home of Service<br>Singh, Akanksha; Ramakrishna Mission Home of Service<br>Singh, Divyanshu; Kiran Society for Rehabilitation and Education of Children with Disabilities<br>Singh, Krishna; Ramakrishna Mission Home of Service<br>Singh, Navin; Kiran Society for Rehabilitation and Education of Children with Disabilities<br>Singh, Neeraj; Kiran Society for Rehabilitation and Education of Children with Disabilities<br>Singh, Rishi; Kiran Society for Rehabilitation and Education of Children with Disabilities<br>Singh, Shyam; Ramakrishna Mission Home of Service<br>Srivastava, Rakesh; Kiran Society for Rehabilitation and Education of Children with Disabilities<br>Srivastava, Sandeep; Kiran Society for Rehabilitation and Education of Children with Disabilities

	Srivastava, Sanjeev; Ramakrishna Mission Home of Service<br>Yadav, Gopal; Kiran Society for Rehabilitation and Education of Children with Disabilities<br>Yadav, Preeti; Kiran Society for Rehabilitation and Education of Children with Disabilities<br>Yadav, Sheshnath; Ramakrishna Mission Home of Service<br>Yadav, Sujata; Ramakrishna Mission Home of Service<br>Marschik, Peter; University Medical Center Göttingen, Department of Child and Adolescent Psychiatry and Psychotherapy; Medical University of Graz, Division of Phoniatrics
**Primary Subject Heading**:	Public health
Secondary Subject Heading:	Paediatrics, Neurology, Health policy
Keywords:	Paediatric neurology < NEUROLOGY, Community child health < PAEDIATRICS, Developmental neurology & neurodisability < PAEDIATRICS, PREVENTIVE MEDICINE, PUBLIC HEALTH

SCHOLARONE™
Manuscripts

I, the Submitting Author has the right to grant and does grant on behalf of all authors of the Work (as defined in the below author licence), an exclusive licence and/or a non-exclusive licence for contributions from authors who are: i) UK Crown employees; ii) where BMJ has agreed a CC-BY licence shall apply, and/or iii) in accordance with the terms applicable for US Federal Government officers or employees acting as part of their official duties; on a worldwide, perpetual, irrevocable, royalty-free basis to BMJ Publishing Group Ltd ("BMJ") its licensees and where the relevant Journal is co-owned by BMJ to the co-owners of the Journal, to publish the Work in this journal and any other BMJ products and to exploit all rights, as set out in our licence.

The Submitting Author accepts and understands that any supply made under these terms is made by BMJ to the Submitting Author unless you are acting as an employee on behalf of your employer or a postgraduate student of an affiliated institution which is paying any applicable article publishing charge ("APC") for Open Access articles. Where the Submitting Author wishes to make the Work available on an Open Access basis (and intends to pay the relevant APC), the terms of reuse of such Open Access shall be governed by a Creative Commons licence – details of these licences and which Creative Commons licence will apply to this Work are set out in our licence referred to above.

Other than as permitted in any relevant BMJ Author's Self Archiving Policies, I confirm this Work has not been accepted for publication elsewhere, is not being considered for publication elsewhere and does not duplicate material already published. I confirm all authors consent to publication of this Work and authorise the granting of this licence.

For peer review only

Enhancing early detection of neurological and developmental disorders and provision of intervention in low-resource settings in Uttar Pradesh, India: Study protocol of the G.A.N.E.S.H. programme

Moreno Toldo,[1a] Swami Varishthananda,[2a] Christa Einspieler,[3a*] Neeraj Tripathi,[1] Anshu Singh,[2] Surendra K. Verma,[1] Kanchan Vishwakarma,[2] Dajie Zhang,[3,4,5] Agyeya Dwivedi,[1] Ritika Gupta,[2] Sanjay Karn,[2] Nirmal Kerketta,[1] Ram Narayan,[1] Karuna Nikam Singh,[6] Sumitra Rani,[2] Akanksha Singh,[2] Divyanshu Singh,[1] Krishna P. Singh,[2] Navin Singh,[1] Neeraj Singh,[1] Rishi Singh,[1] Shyam P. Singh,[2] Rakesh Srivastava,[1] Sandeep Srivastava,[1] Sanjeev Srivastava,[2] Gopal Yadav,[1] Preeti Yadav,[1] Sheshnath Yadav,[2] Sujata Yadav,[2] Peter B. Marschik[3,4,5,7]

[a] These authors contributed equally to the present manuscript (shared first co-authorship)

***Correspondence to**: Dr. Christa Einspieler, Research Unit interdisciplinary Developmental Neuroscience (iDN), Division of Phoniatrics, Medical University of Graz, Auenbruggerplatz 26, 8036 Graz, Austria; E-mail: christa.einspieler@medunigraz.at; Phone: +43 316 385-0

Authors' names and affiliations

Moreno Toldo, Kiran Society for Rehabilitation and Education of Children with Disabilities, Varanasi, India

Swami Varishthananda, Ramakrishna Mission Home of Service, Varanasi, India

Christa Einspieler, Research Unit iDN (interdisciplinary Developmental Neuroscience), Division of Phoniatrics, Medical University of Graz, Graz, Austria

Neeraj Tripathi, Kiran Society for Rehabilitation and Education of Children with Disabilities, Varanasi, India

Anshu Singh, Ramakrishna Mission Home of Service, Varanasi, India

Surendra K. Verma, Kiran Society for Rehabilitation and Education of Children with Disabilities, Varanasi, India

Kanchan Vishwakarma, Ramakrishna Mission Home of Service, Varanasi, India

Dajie Zhang, Department of Child and Adolescent Psychiatry and Psychotherapy, University Medical Center Goettingen, Goettingen, Germany; Research Unit iDN (interdisciplinary

For peer review only

For peer review only - http://bmjopen.bmj.com/site/about/guidelines.xhtml

Developmental Neuroscience), Division of Phoniatrics, Medical University of Graz, Graz, Austria; Leibniz Science Campus Primate Cognition, Goettingen, Germany

Agyeya Dwivedi, Kiran Society for Rehabilitation and Education of Children with Disabilities, Varanasi, India

Ritika Gupta, Ramakrishna Mission Home of Service, Varanasi, India

Sanjay Karn, Ramakrishna Mission Home of Service, Varanasi, India

Nirmal Kerketta, Kiran Society for Rehabilitation and Education of Children with Disabilities, Varanasi, India

Ram Narayan, Kiran Society for Rehabilitation and Education of Children with Disabilities, Varanasi, India

Karuna Nikam Singh, Joints n Motion (JnM) Academy, Mumbai, India

Sumitra Rani, Ramakrishna Mission Home of Service, Varanasi, India

Akanksha Singh, Ramakrishna Mission Home of Service, Varanasi, India

Divyanshu Singh, Kiran Society for Rehabilitation and Education of Children with Disabilities, Varanasi, India

Krishna P. Singh, Ramakrishna Mission Home of Service, Varanasi, India

Navin Singh, Kiran Society for Rehabilitation and Education of Children with Disabilities, Varanasi, India

Neeraj Singh, Kiran Society for Rehabilitation and Education of Children with Disabilities, Varanasi, India

Rishi Singh, Kiran Society for Rehabilitation and Education of Children with Disabilities, Varanasi, India

Shyam P. Singh, Ramakrishna Mission Home of Service, Varanasi, India

Rakesh Srivastava, Kiran Society for Rehabilitation and Education of Children with Disabilities, Varanasi, India

Sandeep Srivastava, Kiran Society for Rehabilitation and Education of Children with Disabilities, Varanasi, India

Sanjeev Srivastava, Ramakrishna Mission Home of Service, Varanasi, India

Gopal Yadav, Kiran Society for Rehabilitation and Education of Children with Disabilities, Varanasi, India

For peer review only

For peer review only - http://bmjopen.bmj.com/site/about/guidelines.xhtml

Preeti Yadav, Kiran Society for Rehabilitation and Education of Children with Disabilities, Varanasi, India

Sheshnath Yadav, Ramakrishna Mission Home of Service, Varanasi, India

Sujata Yadav, Ramakrishna Mission Home of Service, Varanasi, India

Peter B. Marschik, Department of Child and Adolescent Psychiatry and Psychotherapy, University Medical Center Goettingen, Goettingen, Germany; Research Unit iDN (interdisciplinary Developmental Neuroscience), Division of Phoniatrics, Medical University of Graz, Graz, Austria; Leibniz Science Campus Primate Cognition, Goettingen, Germany; Center of Neurodevelopmental Disorders (KIND), Department of Women's and Children's Health, Karolinska Institutet, Stockholm, Sweden

For peer review only

Keywords: cerebral palsy; development; general movement assessment; low resource setting; neurodevelopmental disorders

Word count (excluding, title page, references, figures, tables): 6,584

For peer review only - http://bmjopen.bmj.com/site/about/guidelines.xhtml

ABSTRACT

Introduction: Around 9% of India's children under 6 are diagnosed with neurodevelopmental disorders. Low-resource, rural communities often lack programmes for early identification and intervention. The Prechtl General Movement Assessment (GMA) is regarded as the best clinical tool to predict cerebral palsy in infants < 5 months. In addition, children with developmental delay, intellectual disabilities, late detected genetic disorders or autism spectrum disorder show abnormal general movements (GMs) during infancy. G.A.N.E.S.H. aims to: (a) provide evidence as to whether community health workers can support the identification of infants at high-risk for neurological and developmental disorders and disabilities, (b) monitor further development in those infants, and (c) initiate early and targeted intervention procedures.

Methods: This 3-year observational cohort study will comprise at least 2,000 infants born across four districts of Uttar Pradesh, India. Community health workers, certified for GMA, video record and assess the infants' GMs twice, i.e. within 2 months after birth and at 3-5 months. In case of abnormal GMs and/or reduced motor optimality scores, infants are further examined by a paediatrician and a neurologist. If necessary, early intervention strategies (treatment as usual) are introduced. After paediatric and neurodevelopmental assessments at 12-24 months, outcomes are categorised as normal or neurological/developmental disorders. Research objective 1: to relate the GMA to the outcome at 12-24 months. Research objective 2: to investigate the impact of predefined exposures. Research objective 3: to evaluate the inter-scorer agreement of GMA.

Ethics and dissemination: G.A.N.E.S.H. has received clearance from the Indian Government Chief Medical Officers of Varanasi and Mirzapur and was approved by the Ramakrishna Mission Home of Service in Varanasi. GMA is a worldwide used diagnostic tool, approved by the Ethics Committee of the Medical University of Graz, Austria (27-388 ex 14/15). Apart from peer-reviewed publications, we are planning to deploy G.A.N.E.S.H. in other vulnerable settings.

For peer review only

Strengths and limitations of this study

- G.A.N.E.S.H. includes the application of a non-intrusive, highly reliable assessment tool (the Prechtl General Movement Assessment, GMA) to assist early identification of a high

For peer review only - http://bmjopen.bmj.com/site/about/guidelines.xhtml

risk for cerebral palsy and other neurological and developmental disorders in a low-income geographical cohort.

- G.A.N.E.S.H. aims to provide evidence as to whether a community-based identification and intervention programme facilitates early detection of neurological and developmental disorders and disabilities.
- Measurement biases may occur because blinding is not possible: it has taken the medical doctors and other staff members involved 15 years to win the vulnerable population's trust, which is the basis for conducting a study as outlined here.
- Higher-order impacts may be underestimated as a follow-up period of 24 months may be too short because certain neurological and developmental disorders cannot be diagnosed with absolute certainty at this age.
- Developmental delay in particular, may occur after weaning from breastfeeding because of undernourishment, and might therefore not be detected by means of GMA during the first months of life.

For peer review only

For peer review only - http://bmjopen.bmj.com/site/about/guidelines.xhtml

INTRODUCTION

Background and problem context

India has the world's largest birth ratio (about 26 million per year) and is experiencing dramatic improvements in infant and child survival, although neonatal and infant mortality rates are still high at 30 and 41 per 1,000 live births, respectively.[1] With a population of more than 199 million (ca. 16% of the national population), Uttar Pradesh is the most populous state in India. The 2011 census[2] showed that 59 million people in Uttar Pradesh live below the poverty line. The state's maternal mortality rate is the second highest in India with 285 maternal deaths per 100,000 live births. The under-five mortality rate is 90 per 1,000 live births.[3] Lack of antenatal care, unsafe child births, undernourishment, and lack of access to health care infrastructure are among the main risk factors for maternal and child morbidity and mortality. Neurological and developmental disorders and disabilities in particular compromise the attainment of full social and economic potential at family and community levels. The rate of occurrence of neurodevelopmental disorders ranges from 2.9% to 18.7% in 2- to 9-year-old children across five Indian regions, and its all-site pooled estimate is 9.2% for children younger than 6 years.[4] In other words, almost one in eleven children has at least one of the following impairments: neuromuscular disorders and cerebral palsy, epilepsy, intellectual disability, speech and language disorders, hearing and/or vision impairment, or autism.[4] Pooled estimates are unaffected by gender or rural/urban residence but increase by up to 3 percentage points when adjusted for national rates of low birth weight and/or stunting.[4] The overall pooled prevalence of cerebral palsy per 1,000 children in India is 2.95 (95% CI 2.03 to 3.88).[5]

Apart from low birth weight (<2500g) and preterm birth (< 37 weeks' gestation), non-institutional delivery, a history of perinatal asphyxia (delayed cry and difficult breathing at birth), neonatal illness requiring hospitalisation, postnatal brain infections, and stunting are significantly associated with neurological and developmental disorders and disabilities.[4,5] Several of these risk factors could be averted through public health intervention. There is strong evidence that early intervention improves functional outcomes for infants with neurological and developmental disorders and is economically cost-effective as it reduces the rate and severity of later impairments.[6,7] Early identification of neurodevelopmental impairment is therefore vital for ensuring timely referral to the limited resources provided for impoverished children. However,

For peer review only - http://bmjopen.bmj.com/site/about/guidelines.xhtml

low-income communities, which are most vulnerable to neurological and developmental disorders, often lack programmes for early identification and intervention.[8]

Government programmes such as India's Integrated Child Developmental Services provide supplementary food, health and nutrition education, health check-ups, growth monitoring and child-specific services like immunisation. But to this day only 20% to 30% of people from marginalised groups access these services.[9] Non-governmental organisations, and especially their community health workers, are indispensable for improving the access to early identification and intervention programmes since they work hand in hand with Accredited Social Health Activists (ASHAs), Auxiliary Nurse Midwives (ANMs) and Anganwadi Workers (AWWs), all of whom are part of India's public health care system. It is therefore of utmost importance that community health workers acquire relevant knowledge and skills to be able to recognise early markers for neurological and developmental disorders and refer infants in need to paediatric and neurological examinations. This goal has been supported by the Bill & Melinda Gates Foundation with a Grand Challenges Explorations grant for the development of smartphone-based applications as assistive tools.[10]

About the organisations involved

The impact is assessed by two non-governmental organisations, Kiran Society (KS) and Ramakrishna Mission (RKM), in ten blocks across four districts in the south east of Uttar Pradesh, India.

Kiran Society (KS) is a non-profit organisation situated near Varanasi (also known as Benares) on the banks of the river Ganges in Uttar Pradesh. Since 1990, KS has sought to empower marginalised groups and children with disabilities through rehabilitation, education, vocational training, social integration, and advocacy (www.kiranvillage.org). KS offers institution- and community-based rehabilitation services providing neurological examinations and interventions, physio-, occupational-, speech/language- and psychotherapy as well as corrective orthopaedic aids and appliances. Based on the WHO's community-based rehabilitation matrix and on three schemes, KS' community-based rehabilitation unit currently provides services for 200,000 people in the districts of Varanasi and Mirzapur. One of these schemes is the **G**eneral Movement

For peer review only - http://bmjopen.bmj.com/site/about/guidelines.xhtml

Assessment in **N**eonates for **E**arly Identification and Intervention, **S**ocial Support and **H**ealth Awareness (https://kiranvillage.org/?page_id=3450): G.A.N.E.S.H. was launched in 2018.

Ramakrishna Mission (RKM) is a worldwide organisation founded in 1900. Its Varanasi branch, the RKM Home of Service, has a 230-bed hospital, nine indoor wards and 15 outpatient departments with various diagnostic units. Supported by the WHO, the European Commission, the British Medical Association and others, RKM Varanasi launched its Health Promotion Programme in 2000. Its main objectives are health education, primary health care services and training of community-based personnel in Varanasi and nearby districts. Its 15 actions and research projects include the following three, which are particularly relevant for G.A.N.E.S.H.: (a) child health care education (esp. multimedia presentations on feeding practices, hygiene and sanitation); (b) training community health workers and traditional birth attendants in promoting mother and child health; and (c) mobile mother and child health clinics along with family planning and immunisation interventions. Since 2009, RKM has provided antenatal care and gynaecological services performed by mid-level women health workers in rural areas around Varanasi.

Both organisations' community health workers use mobile tools to pursue a broad range of health education goals including subjects such as vaccination, nutrition, hygiene and sanitation, but most importantly, they provide regular personal care. In addition, both organisations assist the most socio-economically disadvantaged families (especially pregnant women and lactating mothers, infants and young children) in terms of food supplements containing vitamins, iron and calcium.

Project overview and aims

The present observational study is being carried out in a population-based cohort of underprivileged infants in four districts of Uttar Pradesh, India. We are assessing the motor repertoire of individuals aged 1 to 5 months and its association with (a) demographic, pre- and perinatal risk factors, and (b) the neurodevelopmental outcome in the second year of life. The study is conducted by the two non-governmental organisations portrayed above, which have long

For peer review only

For peer review only - http://bmjopen.bmj.com/site/about/guidelines.xhtml

worked together and with governmental organisations in the target area. Disadvantaged families know that KS and RKM provide assistance in case of need.

The staff involved in both organisations received basic and advanced training in the Prechtl General Movement Assessment (GMA)[11], a diagnostic tool for early assessment of the integrity of the young nervous system. Training was provided by a licensed tutor of the GM Trust (C.E.), who also supervises the GMA and data evaluation during the project. Infants at high-risk for neurological and developmental disorders and disabilities are further examined by a paediatrician at RKM (S.V.) and a neurologist at KS (M.T.), who have worked together closely for more than 15 years. Another paediatrician is volunteering to assess infants and children in paediatric camps. All three of them are GMA-trained. KS has a special Medical Fund to guarantee that infants in need receive further diagnostic examination such as EEG, X-ray, MRI, or even genetic testing. If indicated, early physiotherapy is provided by KS staff, while both KS and RKM's teams provide basic health education and counseling.

We expect G.A.N.E.S.H. to improve health outcomes in children living in the target area. The main aims of this 3-year project are to (a) train community health workers to contribute to the identification of infants at high-risk for neurological and developmental disorders early on; (b) monitor further assessments in those infants; and (c) initiate early and targeted intervention procedures (Table 1; Figure 1). Early identification is based on detailed assessments of the spontaneous motor behaviour[11] of all eligible infants born in eight Varanasi villages, 19 Mirzapur villages, one Sonbhadra cluster of villages and one Azamgarh cluster of villages. Research objective 1 is to relate the quantity and quality of movement and postural patterns – which will be determined by means of the Prechtl GMA[11] at 1 to 5 months – to the outcome at 12 to 24 months. Outcome will be assessed by paediatric and neurological examination (including the Hammersmith Infant Neurological Examination[12]) as well as developmental testing (Trivandrum Developmental Screening Charts[13]). Research objective 2 is to investigate whether a number of predefined exposures such as (a) the access to and frequency of antenatal care, (b) maternal micronutrient deficit during pregnancy, (c) safety of child birth, (d) low birth weight (< 2500g) and undernourishment of the infant, and (e) delayed cry at birth are associated with

For peer review only - http://bmjopen.bmj.com/site/about/guidelines.xhtml

movements and postures assessed at 1 to 5 months. Research objective 3 is to examine inter-scorer agreement between community health workers and GMA experts.

METHODS AND ANALYSES

Study design

Observational cohort study which follows the Strengthening the Reporting of Observational Studies in Epidemiology (STROBE) guidelines.

Study setting and profile

In 2019, G.A.N.E.S.H. was implemented in 29 (cluster of) villages in ten blocks of four districts of Uttar Pradesh, where the burden of poverty and undernutrition is particularly high: the Varanasi district (roughly 3,700,000 inhabitants; 1,535 km^2; eight villages in the Kashi Vidyapeeth block, which is covered by KS); the Mirzapur district (roughly 2,500,000 inhabitants; 4,521 km^2; twelve villages in the Majhawan and Shikhar blocks, likewise covered by KS; seven villages or cluster of villages in the Chhanbey, Haliya, Jamalpur, Lalganj, Majhawan, Pahari and Patehara blocks, covered by RKM); the Sonbhadra district (roughly 1,800,000 inhabitants; 6,788 km^2; one cluster of villages in the Robertsganj block, covered by RKM), and the Azamgarh district (roughly 4,600.000 inhabitants; 4,054 km^2; one cluster of villages in the Lalganj block, covered by RKM).

Eastern Uttar Pradesh (particularly the districts of Mirzapur and Sonbhadra) has one of the lowest health indicators of India's eight socioeconomically backward states, also referred to as Empowered Action Group (EAG) states. Poor health is characterised by lack of nutrition, chronic diseases and a high prevalence of infectious diseases resulting not only in high mortality and morbidity but also in an increase of poverty due to out-of-pocket expenses and lost earnings.[14] The main sources of livelihood are agriculture, carpet weaving and pottery, but youth unemployment is high. According to the latest census from 2011[2], the literacy rate then was between 64% in the Sonbhadra district and 75% in the Varanasi district, with a 15 to 20% lower literacy rate in women.

For peer review only - http://bmjopen.bmj.com/site/about/guidelines.xhtml

The target area is characterised by a lack of trained medical staff willing to work in rural areas and a lack of health care infrastructure. Most of the care is provided by the family. Families who seek treatment will often turn to non-allopathic providers such as practitioners of Indian traditional medicine and quacks (i.e. unqualified and unskilled health practitioners pretending to have medical skills).[14] The lack of neurodevelopmental health professionals, particularly in rural areas, places specialist neurodevelopmental care out of the reach of most people. The G.A.N.E.S.H. programme addresses this critical issue and seeks to provide healthcare to families in need.

The target population of G.A.N.E.S.H. comprises approximately 50,000 people. We aim to assess a cohort of at least 2,000 infants born in the first and second years of the study (2019-2020) and collect their outcome data during the second and third years of the study (2020-2021). Due to the COVID-19 pandemic, data collection is deferred since March 15, 2020 and the timeline of the study will be re-scheduled.

In order to discuss the procedure and aims of the G.A.N.E.S.H programme, community health workers of KS and RKM arranged meetings with ASHAs and ANMs in each block. ANMs are village-based female health workers establishing the first contact between the community and health services; in remote areas, ANMs also conduct home deliveries;[15] usually each ANM is supported by four to five ASHAs. ASHAs are local women trained as health educators and appointed by the Ministry of Health and Family Welfare of India. Their tasks include trying to persuade women to give birth in hospitals, taking children to immunisation clinics, encouraging family planning, treating basic illnesses and injuries with first aid, and improving village sanitation.[15]

Each community health worker involved in the project is linked with one ANM and will be informed by them about each live birth in her village. Every Wednesday and Saturday, the government offers free services in primary health centres in our target areas, where ANMs and ASHAs check the infants' weight and growth, promote and assist with breastfeeding and other nutritional aspects, and vaccinate. Community health workers linked to G.A.N.E.S.H. can also recruit mothers with newborn and young infants there. The community health worker approaches the parent(s) within 4 weeks after delivery and explains the G.A.N.E.S.H. project in detail,

For peer review only - http://bmjopen.bmj.com/site/about/guidelines.xhtml

including, for example, mutual commitments. He/she hands out leaflets with relevant information provided in words (Hindi) and pictures. Parents/caregivers are assured of confidentiality and anonymity in reports and publications generated as part of G.A.N.E.S.H. Participation is voluntary, and families are assured that refusing to participate will not affect their access to care. If parents agree to participate, their (written) consent is obtained. Illiterate parents are informed in the presence of a literate witness and consent with their finger-print.

Participants

The birth rate in the target area varies from 19.9 to 28.7 per 1,000.[2] With a population of 50,000 we expect to see 1,000 to 1,500 newborns per year. Apart from living in the target area, inclusion criteria comprise all infants younger than completed 5 months of age irrespective of gender, family background, medical history and current health status. Infants older than 5 months of age are excluded.

The G.A.N.E.S.H. project is perceived by the communities as part of the ongoing services, which have been provided by KS and RKM for decades. More than 90% of newborn infants are brought to the free services in the primary health centers mentioned above, or, alternatively, the community health workers are welcomed at the family home. As part of the Indian culture, after having given birth, young mothers may move to the grandmother's home, possibly in another district. As the majority of the maternal grandparents also live in the same region, the number of moves to other regions is extremely small. Altogether, the number of families not willing to participate in the G.A.N.E.S.H. project is negligibly small.

Variables

After the recruitment of the infant, a community health worker collects the following data (i.e. exposure variables): demographical data including the parents' age; socioeconomic background and caste; maternal medical condition especially during pregnancy and delivery; maternal nutritional status; access to and frequency of antenatal care; site of delivery indicating safety of child birth; complications during labour; the infant's gestational age at birth and birth weight; neonatal complications including a delayed cry at birth (which indicates asphyxia). Frequency of

For peer review only - http://bmjopen.bmj.com/site/about/guidelines.xhtml

antenatal care, gestational age, birth weight, and parental age are continuous data; all other data are categorical.

At 1 to 5 months we assess the following variables: (a) GMs: From birth to the end of the second month postterm age, we assess whether GMs are normal, poor repertoire, or cramped-synchronised[11] (categorical data). From the beginning of the third month to the end of the fifth month postterm age, we assess fidgety movements as normal, abnormal or absent[16] (categorical data) and (b) the motor optimality score[17] (MOS; ordinal data) and its five subscores (ordinal data) as well as the number of normal and abnormal movement and postural patterns (continuous data); (c) clinical data include status of breastfeeding and the infant's medical condition since birth such as fever, diarrhea (categorical data); (d) weight (measured with a portable weighing scale), length (measured on an infantometer), and head circumference (measured with a tape measure extending from the middle of the forehead to the farthest part in the rear of the head) are given as Z-scores, weight-for-age Z-scores, and weight-for-length Z-scores (continuous data).

The outcome at 12 to 24 months is either normal or classified as neurological and developmental disorders including cerebral palsy, epilepsy, (orthopaedic) congenital malformations, muscular dystrophy and spinal muscular atrophy, developmental delay, genetic disorders, visual impairment, hearing impairment, or a high risk for autism spectrum disorder (without the list being exhaustive; categorical data). The scores of the Hammersmith Infant Neurological Examination (HINE)[12] are ordinal data. In addition, each child's nutritional status is assessed and categorised as normal, underweight, wasted (weight-for-age Z-scores that are more than 2 standard deviations below the median of the WHO child growth reference standards), or stunted (with height-for-age Z-scores that are more than 2 standard deviations below the median of the WHO child growth reference standards). Head circumference Z-scores (continuous data) will also be documented.

Data sources and measurement

Exposure variables are obtained through a semi-structured interview and from the medical records of delivery.

For peer review only - http://bmjopen.bmj.com/site/about/guidelines.xhtml

Motor behaviour at 1 to 5 months is assessed by means of the Prechtl General Movement Assessment (GMA),[11] which is widely regarded as the best clinical tool to predict cerebral palsy in infants under 5 months[18] and has proved invaluable for contributing to identify children at risk for developmental delay,[11] cognitive dysfunction,[19] intellectual disabilities,[20,21] speech and language disorders,[22] and autism spectrum disorders[23]. GMA is a diagnostic tool for an early assessment of the integrity of the young nervous system based on observation of young infants' spontaneous movements. It is entirely non-intrusive and extremely time-and cost-efficient. For a reliable assessment (kappa values ranging from 0.88 to 0.92)[11,17], a 2- to 5-minute video of the infant is recorded following a number of basic principles (infant lying in supine position, comfortably dressed, not crying, etc.).[11] Performed by trained assessors, the summary estimates for sensitivity and specificity of GMA are 0.98 (95% CI: 0.74–1.00) and 0.91 (95% CI: 0.83–0.93), respectively.[24] GMA is ideal for the neurological assessment of young infants, especially in low- and middle-income countries that struggle to provide expensive medical equipment for diagnoses.[25] From birth to the end of the second month postterm age, normal general movements (GMs) include a fluctuating sequence of arm, neck, trunk and leg movements. They wax and wane with changing intensity, speed and range of motion. Their onset and ending is typically gradual and smooth. Changing of direction and rotations across the trunk and limb axes are fluent and elegant.[11] From 3 to 5 months, GMs appear as fidgety movements with a very small amplitude and moderate speed of shoulders, wrists, hips and ankles in all directions and of variable acceleration.[11] Most infants will develop normally if fidgety movements are present and normal, especially if they co-occur with other smooth and fluent age-specific movement patterns.[11,16,19] The neural mechanisms of GMs are specific neural networks, central pattern generators (CPG), which are most probably located in the brain stem. We believe that the CPGs for GMs are constantly modulated by more rostral parts of the brain. The resulting variable movements in turn modulate the CPG-activity by variable sensory feedback.[11,19] If a structural impairment of the young nervous system becomes functional, supra-spinal projections are impaired and reduce the modulation of CPG activity, resulting in less variable GMs.[19] Before 3 months of age, abnormal GMs are described as: (i) 'poor repertoire' (the sequence of movements and the intensity, speed and range of motion lack variability) or (ii) 'cramped-synchronised' (the limb and trunk muscles appear rigid and contract almost simultaneously, then relax almost simultaneously).[11] As a sign of functional impairment, fidgety movements can be (iii) 'abnormal'

For peer review only - http://bmjopen.bmj.com/site/about/guidelines.xhtml

(exaggerated in amplitude and speed), (iv) sporadically present (not exceeding 3 seconds[26]) or (v) missing altogether at a postterm age of 9 to 16 weeks; (iv) and (v) are summarised as 'absent fidgety movements'.[11,16,26] Summary estimates of 0.98 for sensitivity and 0.91 for specificity make cramped-synchronised GMs followed by absent fidgety movements very reliable early markers of spastic cerebral palsy.[16-18,24]

The motor repertoire of infants aged 3 to 5 months consists of fidgety movements as well as other movement patterns such as swiping, wiggling-oscillating limb movements, kicking, arm and leg movements to the midline, fiddling, leg lifts, or arching.[17,27]The detailed GMA for 3- to 5-month-old infants comprises the following five subcategories: (i) temporal organisation and quality of fidgety movements; (ii) quality of other observed movement patterns; (iii) age-adequate movement repertoire; (iv) postural patterns; and (v) movement character. Adding the scores of the five subcategories reveals the motor optimality score (MOS)[11,17] with a maximum of 28 (i.e. best possible performance) and a minimum of 5. An MOS ranging from 25 to 28 is considered to be optimal; scores from 20 to 24 are mildly reduced; and an MOS below 20 requires intervention. A score below 9 indicates a very high risk for neurodevelopmental disabilities, especially for non-ambulatory cerebral palsy.[17] Various studies revealed that a reduced MOS was associated with later motor,[17,28-30] cognitive,[29,31] and language[22,29] impairments. A recent worldwide study on 468 children later diagnosed with cerebral palsy shows that such a detailed assessment of early movements and postures provides insight into a child's later functional capabilities and impairment.[17] Intra-observer reliability is high with intra-class correlation coefficients ranging from 0.80 to 0.98[17,28] and Cohen Kappa ranging from 0.87 to 0.91[30].

In February and November 2018, KS and RKM staff involved in G.A.N.E.S.H. (medical doctors, physiotherapists, medical social workers, community-based rehabilitation field workers, special educators, and one psychologist) received training from C.E.. They are now GM Trust-certified (basic and advanced level; www.general-movements-trust.info) to perform GMA. As a side note: for better readability we have summarised the professions of medical social workers, community-based rehabilitation field workers and special educators under the term 'community health workers' throughout the manuscript.

For peer review only - http://bmjopen.bmj.com/site/about/guidelines.xhtml

After parental consent has been received, a GMA-trained KS/RKM staff member videotapes the infant's GMs according to GMA standards (the infant shows 2 to 5 minutes of active wakefulness, must not be crying, fussy or sucking on a pacifier, and must be lying in supine position without manipulation).[11] Video-recordings are preferably at two points in time: (a) during the first 2 months of life; and (b) during the fidgety movement period between the end of the 2nd month and the end of the 5th month postterm age (corrected for preterm delivery). Infants are videotaped in the parents' home, in health camps, or in a primary health centre. The initial evaluation is performed by the GMA-certified staff member who has recorded the video, the second evaluation is performed by the first author (M.T.), and a third evaluation by C.E., who isn't familiar with the medical history of the infant. C.E. supervises the GMA as licensed tutor of the GM Trust. In a pilot sampling approach, we recorded and assessed 400 infants revealing intra-class correlation coefficients ranging from 0.85 to 0.92 between the primary and secondary assessors and the supervisor.

The neurological assessment at 12 to 24 months follows the protocol of the HINE.[12] This method is easily performed and was designed for examining children between 2 and 24 months of age. It includes 26 items that assess cranial nerve function, posture, quality and quantity of movements, muscle tone, and reflexes and reactions. Each item is scored from 0 (minimum) to 3 (maximum score) revealing an overall global score from 0 to 78 (best neurological performance).[12] Both the sensitivity and the specificity are around 0.90.[32]

The Trivandrum Developmental Screening Chart (TDSC)[13] is based on 17 test items from the Bayley Scales of Infant Development (Baroda Norms). It was validated both at hospital and community levels and has a sensitivity of 0.85 and a specificity of 0.91, which makes it a simple, reliable screening tool that can be applied by community health workers.[33] The test-giver draws a vertical line through the chart indicating the actual age of the child. If the child can complete items that are to the left of the line, then there is no delay for that item. If an item lies to the left of the line and the child cannot complete this item, then an item delay is assumed. In this case, a trained assessor at KS will administrate the Bayley Scales of Infant and Toddler Development, Third Edition (cognition, language, motor, social-emotional, and adaptive behaviour domains) with the child. Bayley-III scores exceeding 85 are considered to be normal. In addition, two

For peer review only - http://bmjopen.bmj.com/site/about/guidelines.xhtml

psychologists (one at KS, one at RKM) were trained to identify socio-communicative deficits which, along with repetitive and restricted behaviours, may indicate an increased likelihood of developing autism spectrum disorder.

A potential confounder especially after weaning from breastfeeding (usually after 6 months of age) might be undernutrition. It can be directly attributed to inadequate dietary intake, infection or disease that may affect the child. Lack of sanitation and hygiene, inadequate care, maternal mental health, economic deprivation, and food insecurity might also be factors influencing the child's development. Undernutrition and other external disadvantages hampering thriving will be revealed and documented at all assessment appointments.

Intervention programmes

The infant is referred to a targeted intervention programme if at least one of the following criteria is observed: (a) cramped-synchronised GMs during the first two months; in case of abnormal poor repertoire GMs during the first two months, GMA is repeated at 3 to 5 months, which will determine the further procedure; (b) absence of fidgety movements or exaggerated, abnormal fidgety movements at 3 to 5 months; (c) MOS <20; (d) asymmetries of any kind, especially asymmetric segmental wrist and/or finger movements[17]; (d) body weight and/or head circumference at or below the third age-related percentile on the WHO charts; (e) facial dysmorphism and/or malformations such as congenital talipes equinovarus, cleft palate; (f) acute illness; (g) any other parental concern.

Infants at high-risk for neurological and developmental disorders and disabilities and infants who need medical examination are referred to a paediatrician at RKM (S.V.) and/or a neurologist at KS (M.T.), who have worked together closely for more than 15 years. A second paediatrician is volunteering to assess infants and children in paediatric camps. All three of them are GMA-trained. KS has a special Medical Fund to guarantee that infants in need receive further diagnostic examination such as EEG, X-ray, MRI, or even genetic testing. If indicated, physio-, occupational-, speech/language therapists and special educators treat infants individually at home or at the Parents and Child Care Unit of KS. Since G.A.N.E.S.H. has been launched, every staff

For peer review only - http://bmjopen.bmj.com/site/about/guidelines.xhtml

member involved in interventions has received regular workshops and practical demonstrations of early intervention techniques.

Trained RKM and KS personnel also offers education and counselling on basic health, nutrition and hygiene. Severely anaemic or malnourished mothers and underweight infants are given supplementary nutritious food, vitamins and micronutrients (especially calcium and vitamin D, iron and folic acid tablets) as well as medical care. Infants who need intensive medical treatment are referred to the Ramakrishna Hospital or the district hospitals of Varanasi and Mirzapur. Families in need are invited early on to attend paediatric or rehabilitation camps providing free services, examinations and, if necessary, additional medication.

Bias

Measurement biases may occur because blinding is not possible: it has taken the medical doctors and other staff members involved 15 years to win the vulnerable population's trust, which is the basis for conducting a study as outlined here. In addition, we expect interviewer, recall, and confounder biases. In order to avoid the interviewer bias, the community health worker will follow a standardised interaction protocol with the families. In case the medical record of pregnancy and delivery is not available (lack of antenatal care and home delivery) an eventual recall bias of the mother is difficult to avoid. We will be able to control the confounder bias (e.g. undernutrition), as we will document any recognizable external disadvantages hampering thriving, and compare the Z-values of the anthropometric measurements at birth, 1 to 5 months, and 12 to 24 months.

Study size

Taken an alpha cut-off of 5% and a beta cut-off of 20%, the sample shall involve 1,261 children (i.e. with a pooled incidence of 9.2% neurodevelopmental disorders in Indian children younger than 6 years,[4] and an expected incidence of 7% in the study group, with a birth rate in the target area varying from 19.9 to 28.7 per 1,000[2], that is, we expect to see 1,000 to 1,500 newborns per year, resulting in about 70 to 105 children with neurological and developmental disorders and disabilities) to achieve a predictive power of .95 and an effect-size of .15 using Cohen's f^2. According to our eligibility criteria, we aimed to assess a cohort of at least 2,000 infants born in

For peer review only - http://bmjopen.bmj.com/site/about/guidelines.xhtml

the first and second year of the study. Until March 15, 2020 (13 months after launching the study, before data collection was deferred due to the COVID-19 pandemic), we have successfully recruited 1,300 infants.

Statistical methods

Data will be analysed according to the research objectives of the study using IBM SPSS Statistics in its updated version with significance set at $p<0.05$. A lead biostatistician of the University Medical Center Goettingen, Germany, will provide expert input for the analyses.

Initial descriptive analysis will explore the characteristics and distributions of the variables. Any outlying data will be identified and the missing data explored. Histograms, boxplots and scatter plots, tables of means and SD or medians, interquartile ranges, and cross tabulations will be used as appropriate.

Sensitivity, specificity, and positive and negative predictive values will be determined with 95% CIs. This will be based on the positive / negative 'high risk of neurological and developmental disorders' status on GMs and MOS (at 3 to 5 months) compared with confirmed or suspected neurological and developmental disorders / no neurological and developmental disorders diagnoses (at 12 to 24 months). The association between the MOS (at 3 to 5 months) and the HINE (at 12 to 24 months) will be examined using Spearman's rank-order correlations. To test the predictive power of the MOS-subcategories (polytomous ordinal predictors) on the HINE cut-off outcomes (polytomous ordinal criterion), we will run ordinal regression analysis. The predictive power of abnormal movement and postural patterns (metric predictors) on HINE cut-off outcomes (polytomous ordinal criterion) will be again estimated by means of ordinal regression analysis.

We will perform regression analyses to determine how exposure variables are related to GMs and MOS. General linear model analyses will be performed to understand the effect and interactions of predefined variables and covariates on GMs. In case of multiple subgroup comparisons, we will divide the significant level of 0.05 by the total number of subgroups of the analysis (Bonferroni correction).

For peer review only - http://bmjopen.bmj.com/site/about/guidelines.xhtml

GMs and MOS will be rated by at least one community health worker, the first (M.T.) and the third author (C.E.). Fleiss' kappa will be calculated to assess the agreement among the three scorers when assigning categorical ratings to GMs. Intra-class correlation coefficient (ICC) statistics will be applied to examine the agreement of the MOS among the scorers.

Longer-term outcomes

The listed longer-term outcomes are those we expect to achieve in a follow-up evaluation period of 3 to 5 years, depending on the availability of funds. Details will be determined later, as they can change according to requirements. Long-term outcomes should reflect an improved child health and nutritional status, improved motor, cognitive, and language abilities, a better supply of vision and hearing aids and orthoses, proper management in case of epilepsy, access to MRI, EEG, X-ray and other diagnostic procedures, as well as adequate psychological treatment and counselling – in other words: a proper management of neurological and developmental disorders and disabilities and a strengthened health system.

Patient and Public Involvement

It has not been appropriate to involve infants and their families or the public in the design, or conduct, or reporting, or dissemination plans of G.A.N.E.S.H.

Ethics and dissemination

G.A.N.E.S.H. has received clearance from the Indian Government Chief Medical Officers of Varanasi and Mirzapur and was approved by the Ramakrishna Mission Home of Service in Varanasi. GMA is a worldwide used diagnostic tool, approved by the Ethics Committee of the Medical University of Graz, Austria (27-388 ex 14/15).

After parents/caregivers have received detailed information on G.A.N.E.S.H., they are assured of confidentiality and anonymity in reports and publications generated as part of G.A.N.E.S.H. Participation is voluntary, and families are assured that refusing to participate will not affect their access to care. If parents agree to participate, their (written) consent is obtained. Illiterate parents are informed in the presence of a literate witness and consent with their fingerprint. All study data are stored in secure databases. Paper files are kept in locked filing cabinets, and computer

For peer review only - http://bmjopen.bmj.com/site/about/guidelines.xhtml

and video files are stored on password-protected servers. Only staff members associated with the G.A.N.E.S.H. programme have access to the data.

Results are submitted to peer-reviewed journals and presented at national and international conferences. Beyond that, we aim for G.A.N.E.S.H. to serve as a model for early identification of and intervention in children with (or a high-risk for) neurological and developmental disorders and disabilities, to have a sustainably impact on health care plans for low-income settings. Therefore, dissemination to health care professionals and policy makers is crucial.

All results presented are group data; individual participants are not identifiable. We intend to analyse and publish GMA and MOS results as well as 12-to 24-month outcomes as stand-alone research articles in peer-reviewed journals. In accordance with our main aim to improve the quality and quantity of interactions with beneficiary families, the main research questions (in chronological order) to answer and discuss, are:

1. Is it feasible for trained community health workers to launch G.A.N.E.S.H. and apply GMA in a rural, low-income setting? Key aspects: s*uccess of training, acceptance by ASHAs, ANWs, acceptance in the community, challenges, inter-scorer agreement, frequent staff meetings, expert supervision.* Limitations/risks: *parental refusal, cultural setting in case of male community health worker, migration of trained staff, priority of nutritional aspects over GMA.*
2. Does G.A.N.E.S.H. improve the frequency of home visits for infants and their mothers and the extent and level of counselling for families in need, and does it help to identify abnormal motor behaviour and malnutrition? Key aspects: *identification of abnormal GMs and reduced MOS, parental compliance with further paediatric and neurological examination, launching of targeted family-oriented intervention, targeted health education, provision of food supplementation and micronutrients.* Limitations/risks: *no compliance with further assessment and intervention because early markers are subtle; families see the need to consult a doctor only in case of (frequent) seizures, acute infection, or malformations like cleft palate and congenital talipes equinovarus. The need for prevention and early intervention is difficult to grasp for parents, and is often impeded by cultural hierarchical family structure.*

For peer review only - http://bmjopen.bmj.com/site/about/guidelines.xhtml

3. Are we able to identify early on (especially at 3 to 5 months) children who will have an adverse outcome around 12 to 24 months of age? Key aspects: *predictive value of GMA, sustainable family acceptance and compliance with targeted strategies of early intervention.* Limitations/risks: *the nutritional status, especially after weaning from breastfeeding (second half of the first year of life) might have a greater impact on the development than the integrity of the nervous system determined by GMA.*

Acknowledgements: The authors are grateful to Professor Akmer Mutlu, Hacettepe University Ankara, Turkey for providing teaching material to the physiotherapy unit at Kiran Society; to Mrs. Sikha Sharma for basic insights into the principles of early intervention; to Mr. Amarendra Kumar and Mr. Vijay Pal for their support in community-based rehabilitation; to Dr Ruchi Pandey for her assistance in paediatric camps; to Mrs. Sangeeta J.K., founder and president of Kiran Society, for introducing the community-based rehabilitation approach among Kiran Society's activities and her ongoing support and inspiration; and to Miha Tavcar (www.scriptophil.at) for copy-editing the article.

Contributors: MT, SV and CE conceptualised G.A.N.E.S.H. and conceived the research questions. MT, SV, CE and PBM secured funding for the project and prepared the study protocol with feedback from all collaborators. MT, SVP and CE are principle investigators, PBM is co-investigator. NT and AnS supervise the data collection under the guidance of investigators. SKV, AnS, KV, AkS, and CE developed the data base for the project. DZ provided advice on the statistical design of the study. MT, SV, NT, AnS, KNS, DS, NaS, and RaS supervise interventions applied in G.A.N.E.S.H. All authors read and approved the final protocol.

Funding: This work is supported by Bill & Melinda Gates Foundation (opp1128871), the Austrian Science Fund FWF (P25241, TCS24, KLI811), the GM Trust (WHEP23-8-5-16), the Australian Cerebral Palsy Alliance (SEG00419), the Old Possum's Practical Trust, the Medical Fund of Kiran Society and the Ramakrishna Mission.

For peer review only

Competing interests: The GM Trust supports G.A.N.E.S.H.. CE is a GM Trust tutor for GMA, and PBM is president of the GM Trust (honorary function), both of whom have contributed to G.A.N.E.S.H. on an honorary basis.

Ethics Approval: G.A.N.E.S.H. has received clearance from the Indian Government Chief Medical Officers of Varanasi and Mirzapur and was approved by the Ramakrishna Mission Home of Service in Varanasi. GMA is a worldwide used diagnostic tool, approved for its retrospective and prospective application by the Ethics Committee of the Medical University of Graz, Austria (27-388 ex 14/15).

Provenance and peer review: Not commissioned; externally peer-reviewed.

Data sharing statement: This being a protocol no unpublished data are available.

REFERENCES

1. International Institute for Population Sciences (IIPS) and ICF. National Family Health Survey (NFHS-4), 2015-2016: India. Mumbai: IIPS, 2017. http://rchiips.org/NFHS/NFHS-4Reports/India.pdf (accessed Nov 25, 2019).
2. Census of India 2011. www.censusindia.gov.in (accessed Oct 3, 2019).
3. Annual Health Survey Report, Part I. Office of the Registrar General & Census Commissioner. Ministry of Home Affairs, Government of India, 2013.
4. Arora NK, Nair MKC, Gulati S, *et al.* Neurodevelopmental disorders in children aged 2–9 years: Population-based burden estimates across five regions in India. *PLoS Med* 2018;15:e1002615.
5. Chauhan A, Singh M, Jaiswal N, *et al*. Prevalence of cerebral palsy in Indian children: a systematic review and meta-analysis. *Indian J Pediatr* 2019;doi 10.1007/s12098-019-03024-0.
6. Spittle A, Orton J, Anderson P, *et al*. Early developmental intervention programmes post-hospital discharge to prevent motor and cognitive impairments in preterm infants. *Cochrane Database Syst Rev* 2015;11:CD005495.

For peer review only - http://bmjopen.bmj.com/site/about/guidelines.xhtml

7. Benfer KA, Novak I, Morgan C, *et al*. Community-based parent-delivered early detection and intervention programme for infants at high risk of cerebral palsy in a low-resource country (Learning through Everyday Activities with Parents (LEAP-CP): protocol for a randomized controlled trial. *BMJ Open* 2018;8:e021186.

8. UN World Health Organization (WHO). 58th World Health Assembly: Resolutions and Decisions, Annex. Geneva: World Health Organization 2005. Document WHA58/2005/REC/1. http://apps.who.int/gb/ebwha/pdf_files/WHA58-REC1/english/A58_2005_REC1-en.pdf (accessed at July 15, 2019).

9. Chakrabarti S, Raghunathan K, Alderman H, *et al*. India's Integrated Child Development Services programme; equity and extent of coverage in 2006 and 2016. *Bull World Health Organ* 2019;97:270-282.

10. Marschik PB, Pokorny FB, Peharz R, *et al*. A novel way to measure and predict development: a heuristic approach to facilitate the early detection of neurodevelopmental disorders. *Curr Neurol Neurosci Rep* 2017;17:43. doi 10.1007/s11910-017-0748-8

11. Einspieler C, Prechtl HFR, Bos AF, *et al*. *Prechtl's Method on the Qualitative Assessment of General Movements in Preterm, Term and Young Infants*. London, UK: Mac Keith Press 2004.

12. Haataja L, Mercuri E, Regev R, *et al*. Optimality score for the neurologic examination of the infant at 12 and 18 months of age. *J Pediatr* 1999;135:153-161.

13. Nair MK, George B, Philip E, *et al*. Trivandrum developmental screening chart. *Indian Pediatr* 1991;28:869-872.

14. Arokiasamy P, Gautam A. Neonatal mortality in the empowered action group states of India: trends and determinants. *J Biosoc Sci* 2008;40:183-201.

15. Nimmagadda S, Gopalakrishnan L, Avula R, *et al*. Effects of an mHealth intervention for community health workers on maternal and child nutrition and health services delivery in India: protocol for a quasi-experimental mixed-methods evaluation. *BMJ Open* 2019;9:e025774.

16. Prechtl HFR, Einspieler C, Cioni G, *et al.* An early marker for neurological deficits after perinatal brain lesions. *Lancet* 1997;349:1361-1363.

For peer review only - http://bmjopen.bmj.com/site/about/guidelines.xhtml

17. Einspieler C, Bos AF, Krieber-Tomantschger M, *et al*. Cerebral palsy: early markers of clinical phenotype and functional outcome. *J Clin Med* 2019;8:1616. doi: 10.3390/jcm8101616.

18. Novak I, Morgan C, Adde, L, *et al.* Early, accurate diagnosis and early intervention in cerebral palsy: advances in diagnosis and treatment. *JAMA Pediatr* 2017;171:897-907.

19. Einspieler C, Bos AF, Libertus ME, *et al*. The general movement assessment helps us to identify preterm infants at risk for cognitive dysfunction. *Front Psychol* 2016;7:406. doi 10.3389/fpsyg.2016.00406.

20. Herrero D, Einspieler C, Panvequio Aizawa CY, *et al*. The motor repertoire in 3- to 5-month old infants with Down syndrome. *Res Dev Disabil* 2017;67:1-8.

21. Einspieler C, Marschik PB. Regression in Rett syndrome: Developmental pathways to its onset. *Neurosci Biobehav Rev* 2019,98:320-332.

22. Salavati S, Einspieler C, Vagelli G, *et al*. The association between the early motor repertoire and language development in term children born after normal pregnancy. *Early Hum Dev* 2017;111:30-35.

23. Einspieler C, Sigafoos J, Bartl-Pokorny KD, *et al.* Highlighting the first 5 months of life: general movements in infants later diagnosed with autism spectrum disorder or Rett syndrome. *Res Autism Spectr Dis* 2014;8:286-291.

24. Bosanquet M, Copeland L, Ware R, *et al*. A systematic review of tests to predict cerebral palsy in young children. *Dev Med Child Neurol* 2013;55:418-426.

25. Tomantschger I, Herrero D, Einspieler C, *et al*. The general movement assessment in non-European low- and middle-income countries. *Rev Saude Publica* 2018;52:6. doi: 10.11606/S1518-8787.2018052000332.

26. Einspieler C, Yang H, Bartl-Pokorny KD, *et al.* Are sporadic fidgety movements as clinically relevant as is their absence? *Early Hum Dev* 2015;91:247-252.

27. Einspieler C, Marschik PB, Prechtl HFR. Human motor behaviour. Prenatal origin and early postnatal development. *J Psychol* 2008;216:148-154.

28. Zang FF, Yang H, Han Q, *et al*. Very low birth weight infants in China: the predictive value of the motor repertoire at 3 to 5 months for the motor performance at 12 months. *Early Hum Dev* 2016;100:27-32.

For peer review only

For peer review only - http://bmjopen.bmj.com/site/about/guidelines.xhtml

29. Einspieler C, Utsch F, Brasil P, *et al*. Association of infants exposed to prenatal Zika virus infection with their clinical, neurologic, and developmental status evaluated via the general movement assessment tool. *JAMA Netw Open* 2019a; e187235.

30. Bruggink JL, Einspieler C, Butcher PR, *et al*. Quantitative aspects of the early motor repertoire in preterm infants: do they predict minor neurological dysfunction at school age? *Early Hum Dev* 2009;85:25-36.

31. Butcher PR, van Braeckel K, Bouma A, *et al*. The quality of preterm infants' spontaneous movements: an early indicator of intelligence and behaviour at school age. *J Child Psychol Psychiatry* 2009;50:920-30.

32. Romeo DM, Ricci D, Brogna C, *et al*. Use of the Hammersmith Infant Neurological Examination in infants with cerebral palsy: a critical review of the literature. *Dev Med Child Neurol* 2016;58:240-245.

33. Nair MK, Nair GS, George B, *et al*. Development and validation of Trivandrum Developmental Screening Chart for children aged 0-6 years [TDSC (0-6)]. *Indian J Pediatr* 2913;80:S248-S255.

For peer review only

Table 1: Main procedures and outcome measures

Procedures	Output	
Training community health workers to identify infants at high-risk for neurological and developmental disorders and disabilities early on	(1)	staff trained and certified in basic and advanced GMA
	(2)	agreement (a) between the scorers, (b) between the scorers and a GMA-trained neurologist, and (c) between the scorers and a GMA expert
Monitoring further assessments of infants at high-risk for neurological and developmental disorders and disabilities	(3)	number of infants screened
	(4)	number of infants identified (abnormal age-specific GMs, reduced MOS)
	(5)	number of families supported
	(6)	number of underweight infants
	(7)	number of infants examined by a paediatrician and/or a neurologist; examination results
	(8)	number of (breastfeeding) mothers and infants who received micronutrient supply
	(9)	number of infants who received (financially supported) access to MRI, EEG, X-ray, and laboratory testing
Launching targeted intervention procedures early on.	(10)	number of staff trained in early parent oriented intervention
	(11)	number of infants referred to early intervention
	(12)	number of families referred to further parent training (around the infant's age of 6 to 7 months)

Key: EEG, electroencephalography, GMA, general movement assessment; MOS, motor optimality score; MRI, magnetic resonance imaging

For peer review only - http://bmjopen.bmj.com/site/about/guidelines.xhtml

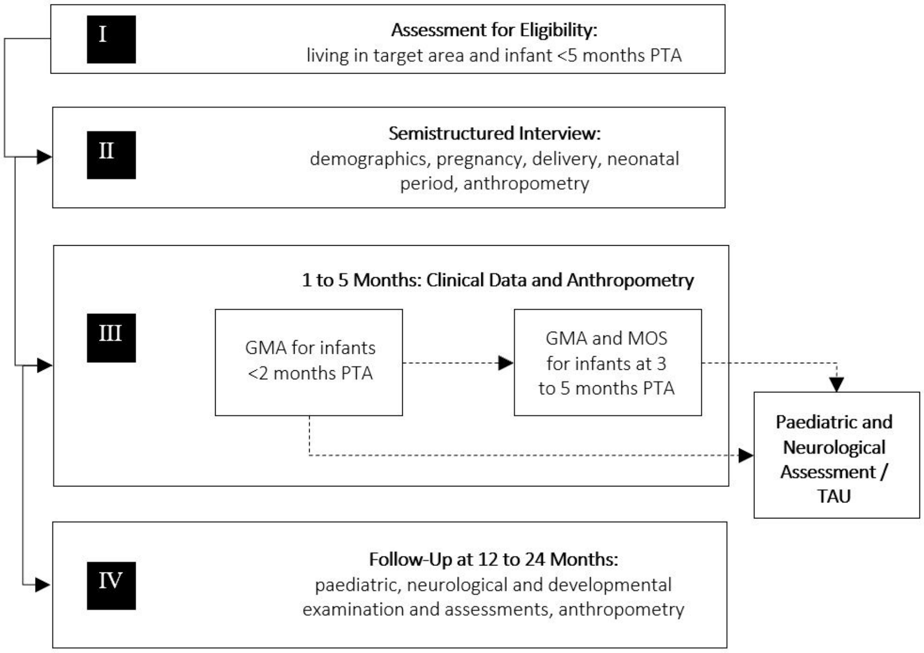

Study design of the G.A.N.E.S.H. programme
Key: GMA, general movement assessment; MOS, motor optimality score; PTA, postterm age; TAU, treatment as usual

140x106mm (192 x 192 DPI)

STROBE Statement—Checklist of items that should be included in reports of ***cohort studies***

	Item No	Recommendation	Page No
**Title and abstract**	1	(*a*) Indicate the study's design with a commonly used term in the title or the abstract	1
		(*b*) Provide in the abstract an informative and balanced summary of what was done and what was found	1
**Introduction**			
Background/rationale	2	Explain the scientific background and rationale for the investigation being reported	3-6
Objectives	3	State specific objectives, including any prespecified hypotheses	6-7
**Methods**			
Study design	4	Present key elements of study design early in the paper	7
Setting	5	Describe the setting, locations, and relevant dates, including periods of recruitment, exposure, follow-up, and data collection	7-9
Participants	6	(*a*) Give the eligibility criteria, and the sources and methods of selection of participants. Describe methods of follow-up	9
		(*b*) For matched studies, give matching criteria and number of exposed and unexposed	
Variables	7	Clearly define all outcomes, exposures, predictors, potential confounders, and effect modifiers. Give diagnostic criteria, if applicable	9-10
Data sources/ measurement	8*	For each variable of interest, give sources of data and details of methods of assessment (measurement). Describe comparability of assessment methods if there is more than one group	10-14
Bias	9	Describe any efforts to address potential sources of bias	15
Study size	10	Explain how the study size was arrived at	15-16
Quantitative variables	11	Explain how quantitative variables were handled in the analyses. If applicable, describe which groupings were chosen and why	9-10
Statistical methods	12	(*a*) Describe all statistical methods, including those used to control for confounding	16-17
		(*b*) Describe any methods used to examine subgroups and interactions	16
		(*c*) Explain how missing data were addressed	16
		(*d*) If applicable, explain how loss to follow-up was addressed	
		(*e*) Describe any sensitivity analyses	16
**Results**			
Participants	13*	(a) Report numbers of individuals at each stage of study—eg numbers potentially eligible, examined for eligibility, confirmed eligible, included in the study, completing follow-up, and analysed	n.a.
		(b) Give reasons for non-participation at each stage	
		(c) Consider use of a flow diagram	
Descriptive data	14*	(a) Give characteristics of study participants (eg demographic, clinical, social) and information on exposures and potential confounders	n.a.
		(b) Indicate number of participants with missing data for each variable of interest	
		(c) Summarise follow-up time (eg, average and total amount)	
Outcome data	15*	Report numbers of outcome events or summary measures over time	n.a.

For peer review only

Main results	16	(*a*) Give unadjusted estimates and, if applicable, confounder-adjusted estimates and their precision (eg, 95% confidence interval). Make clear which confounders were adjusted for and why they were included<br>(*b*) Report category boundaries when continuous variables were categorized<br>(*c*) If relevant, consider translating estimates of relative risk into absolute risk for a meaningful time period	n.a
Other analyses	17	Report other analyses done—eg analyses of subgroups and interactions, and sensitivity analyses	
**Discussion**			
Key results	18	Summarise key results with reference to study objectives	n.a.
Limitations	19	Discuss limitations of the study, taking into account sources of potential bias or imprecision. Discuss both direction and magnitude of any potential bias	
Interpretation	20	Give a cautious overall interpretation of results considering objectives, limitations, multiplicity of analyses, results from similar studies, and other relevant evidence	
Generalisability	21	Discuss the generalisability (external validity) of the study results	
**Other information**			
Funding	22	Give the source of funding and the role of the funders for the present study and, if applicable, for the original study on which the present article is based	19-20

*Give information separately for exposed and unexposed groups.

Note: An Explanation and Elaboration article discusses each checklist item and gives methodological background and published examples of transparent reporting. The STROBE checklist is best used in conjunction with this article (freely available on the Web sites of PLoS Medicine at http://www.plosmedicine.org/, Annals of Internal Medicine at http://www.annals.org/, and Epidemiology at http://www.epidem.com/). Information on the STROBE Initiative is available at http://www.strobe-statement.org.

For peer review only

For peer review only - http://bmjopen.bmj.com/site/about/guidelines.xhtml

BMJ Open

Enhancing early detection of neurological and developmental disorders and provision of intervention in low-resource settings in Uttar Pradesh, India: Study protocol of the G.A.N.E.S.H. programme

Journal:	*BMJ Open*
Manuscript ID	bmjopen-2020-037335.R2
Article Type:	Protocol
Date Submitted by the Author:	01-Oct-2020

Complete List of Authors:	Toldo, Moreno; Kiran Society for Rehabilitation and Education of Children with Disabilities<br>Varishthananda , Swami; Ramakrishna Mission Home of Service<br>Einspieler, Christa; Medical University of Graz, Division of Phoniatrics<br>Tripathi, Neeraj; Kiran Society for Rehabilitation and Education of Children with Disabilities<br>Singh, Anshu; Ramakrishna Mission Home of Service<br>Verma, Surendra; Kiran Society for Rehabilitation and Education of Children with Disabilities<br>Vishwakarma, Kanchan; Ramakrishna Mission Home of Service<br>Zhang, Dajie; University Medical Center Göttingen, Department of Child and Adolescent Psychiatry and Psychotherapy; Medical University of Graz, Division of Phoniatrics<br>Dwivedi, Agyeya; Kiran Society for Rehabilitation and Education of Children with Disabilities<br>Gupta, Ritika; Ramakrishna Mission Home of Service<br>Karn, Sanjay; Ramakrishna Mission Home of Service<br>Kerketta, Nirmal; Kiran Society for Rehabilitation and Education of Children with Disabilities<br>Narayan, Ram; Kiran Society for Rehabilitation and Education of Children with Disabilities<br>Nikam Singh, Karuna; Joints n Motion (JnM) Academy<br>Rani, Sumitra; Ramakrishna Mission Home of Service<br>Singh, Akanksha; Ramakrishna Mission Home of Service<br>Singh, Divyanshu; Kiran Society for Rehabilitation and Education of Children with Disabilities<br>Singh, Krishna; Ramakrishna Mission Home of Service<br>Singh, Navin; Kiran Society for Rehabilitation and Education of Children with Disabilities<br>Singh, Neeraj; Kiran Society for Rehabilitation and Education of Children with Disabilities<br>Singh, Rishi; Kiran Society for Rehabilitation and Education of Children with Disabilities<br>Singh, Shyam; Ramakrishna Mission Home of Service<br>Srivastava, Rakesh; Kiran Society for Rehabilitation and Education of Children with Disabilities<br>Srivastava, Sandeep; Kiran Society for Rehabilitation and Education of Children with Disabilities

	Srivastava, Sanjeev; Ramakrishna Mission Home of Service<br>Yadav, Gopal; Kiran Society for Rehabilitation and Education of Children with Disabilities<br>Yadav, Preeti; Kiran Society for Rehabilitation and Education of Children with Disabilities<br>Yadav, Sheshnath; Ramakrishna Mission Home of Service<br>Yadav, Sujata; Ramakrishna Mission Home of Service<br>Marschik, Peter; University Medical Center Göttingen, Department of Child and Adolescent Psychiatry and Psychotherapy; Medical University of Graz, Division of Phoniatrics
**Primary Subject Heading**:	Public health
Secondary Subject Heading:	Paediatrics, Neurology, Health policy
Keywords:	Paediatric neurology < NEUROLOGY, Community child health < PAEDIATRICS, Developmental neurology & neurodisability < PAEDIATRICS, PREVENTIVE MEDICINE, PUBLIC HEALTH

SCHOLARONE™
Manuscripts

I, the Submitting Author has the right to grant and does grant on behalf of all authors of the Work (as defined in the below author licence), an exclusive licence and/or a non-exclusive licence for contributions from authors who are: i) UK Crown employees; ii) where BMJ has agreed a CC-BY licence shall apply, and/or iii) in accordance with the terms applicable for US Federal Government officers or employees acting as part of their official duties; on a worldwide, perpetual, irrevocable, royalty-free basis to BMJ Publishing Group Ltd ("BMJ") its licensees and where the relevant Journal is co-owned by BMJ to the co-owners of the Journal, to publish the Work in this journal and any other BMJ products and to exploit all rights, as set out in our licence.

The Submitting Author accepts and understands that any supply made under these terms is made by BMJ to the Submitting Author unless you are acting as an employee on behalf of your employer or a postgraduate student of an affiliated institution which is paying any applicable article publishing charge ("APC") for Open Access articles. Where the Submitting Author wishes to make the Work available on an Open Access basis (and intends to pay the relevant APC), the terms of reuse of such Open Access shall be governed by a Creative Commons licence – details of these licences and which Creative Commons licence will apply to this Work are set out in our licence referred to above.

Other than as permitted in any relevant BMJ Author's Self Archiving Policies, I confirm this Work has not been accepted for publication elsewhere, is not being considered for publication elsewhere and does not duplicate material already published. I confirm all authors consent to publication of this Work and authorise the granting of this licence.

For peer review only

Enhancing early detection of neurological and developmental disorders and provision of intervention in low-resource settings in Uttar Pradesh, India: Study protocol of the G.A.N.E.S.H. programme

Moreno Toldo,[a] Swami Varishthananda,[a] Christa Einspieler,[a*] Neeraj Tripathi, Anshu Singh, Surendra K. Verma, Kanchan Vishwakarma, Dajie Zhang, Agyeya Dwivedi, Ritika Gupta, Sanjay Karn, Nirmal Kerketta, Ram Narayan, Karuna Nikam Singh, Sumitra Rani, Akanksha Singh, Divyanshu Singh, Krishna P. Singh, Navin Singh, Neeraj Singh, Rishi Singh, Shyam P. Singh, Rakesh Srivastava, Sandeep Srivastava, Sanjeev Srivastava, Gopal Yadav, Preeti Yadav, Sheshnath Yadav, Sujata Yadav, Peter B. Marschik

[a] These authors contributed equally to the present manuscript (shared first co-authorship)

Dedicated to the late Swami Varishthananda, Ramakrishna Mission Home of Service, Varanasi, India

*Correspondence to: Dr. Christa Einspieler, Professor of Physiology, Research Unit interdisciplinary Developmental Neuroscience (iDN), Division of Phoniatrics, Medical University of Graz, Auenbruggerplatz 26, 8036 Graz, Austria; Phone: +43 316 385-0; E-mail: christa.einspieler@medunigraz.at

Authors and Affiliations

Moreno Toldo, Kiran Society for Rehabilitation and Education of Children with Disabilities, Varanasi, India

Swami Varishthananda, Ramakrishna Mission Home of Service, Varanasi, India

Christa Einspieler, Research Unit iDN (interdisciplinary Developmental Neuroscience), Division of Phoniatrics, Medical University of Graz, Graz, Austria

Neeraj Tripathi, Kiran Society for Rehabilitation and Education of Children with Disabilities, Varanasi, India

Anshu Singh, Ramakrishna Mission Home of Service, Varanasi, India

For peer review only - http://bmjopen.bmj.com/site/about/guidelines.xhtml

Surendra K. Verma, Kiran Society for Rehabilitation and Education of Children with Disabilities, Varanasi, India

Kanchan Vishwakarma, Ramakrishna Mission Home of Service, Varanasi, India

Dajie Zhang, Department of Child and Adolescent Psychiatry and Psychotherapy, University Medical Center Goettingen, Goettingen, Germany; Research Unit iDN (interdisciplinary Developmental Neuroscience), Division of Phoniatrics, Medical University of Graz, Graz, Austria; Leibniz Science Campus Primate Cognition, Goettingen, Germany

Agyeya Dwivedi, Kiran Society for Rehabilitation and Education of Children with Disabilities, Varanasi, India

Ritika Gupta, Ramakrishna Mission Home of Service, Varanasi, India

Sanjay Karn, Ramakrishna Mission Home of Service, Varanasi, India

Nirmal Kerketta, Kiran Society for Rehabilitation and Education of Children with Disabilities, Varanasi, India

Ram Narayan, Kiran Society for Rehabilitation and Education of Children with Disabilities, Varanasi, India

Karuna Nikam Singh, Joints n Motion (JnM) Academy, Mumbai, India

Sumitra Rani, Ramakrishna Mission Home of Service, Varanasi, India

Akanksha Singh, Ramakrishna Mission Home of Service, Varanasi, India

Divyanshu Singh, Kiran Society for Rehabilitation and Education of Children with Disabilities, Varanasi, India

Krishna P. Singh, Ramakrishna Mission Home of Service, Varanasi, India

Navin Singh, Kiran Society for Rehabilitation and Education of Children with Disabilities, Varanasi, India

Neeraj Singh, Kiran Society for Rehabilitation and Education of Children with Disabilities, Varanasi, India

Rishi Singh, Kiran Society for Rehabilitation and Education of Children with Disabilities, Varanasi, India

Shyam P. Singh, Ramakrishna Mission Home of Service, Varanasi, India

Rakesh Srivastava, Kiran Society for Rehabilitation and Education of Children with Disabilities, Varanasi, India

For peer review only

Sandeep Srivastava, Kiran Society for Rehabilitation and Education of Children with Disabilities, Varanasi, India

Sanjeev Srivastava, Ramakrishna Mission Home of Service, Varanasi, India

Gopal Yadav, Kiran Society for Rehabilitation and Education of Children with Disabilities, Varanasi, India

Preeti Yadav, Kiran Society for Rehabilitation and Education of Children with Disabilities, Varanasi, India

Sheshnath Yadav, Ramakrishna Mission Home of Service, Varanasi, India

Sujata Yadav, Ramakrishna Mission Home of Service, Varanasi, India

Peter B. Marschik, Department of Child and Adolescent Psychiatry and Psychotherapy, University Medical Center Goettingen, Goettingen, Germany; Research Unit iDN (interdisciplinary Developmental Neuroscience), Division of Phoniatrics, Medical University of Graz, Graz, Austria; Leibniz Science Campus Primate Cognition, Goettingen, Germany; Center of Neurodevelopmental Disorders (KIND), Department of Women's and Children's Health, Karolinska Institutet, Stockholm, Sweden

Key words: community based rehabilitation; general movement assessment; low-resource setting; motor optimality score; prediction

Word count: 6,911

For peer review only - http://bmjopen.bmj.com/site/about/guidelines.xhtml

ABSTRACT

Introduction: Around 9% of India's children under 6 are diagnosed with neurodevelopmental disorders. Low-resource, rural communities often lack programmes for early identification and intervention. The Prechtl General Movement Assessment (GMA) is regarded as the best clinical tool to predict cerebral palsy in infants < 5 months. In addition, children with developmental delay, intellectual disabilities, late detected genetic disorders or autism spectrum disorder show abnormal general movements (GMs) during infancy. G.A.N.E.S.H. aims to: (a) provide evidence as to whether community health workers can support the identification of infants at high-risk for neurological and developmental disorders and disabilities, (b) monitor further development in those infants, and (c) initiate early and targeted intervention procedures.

Methods: This 3-year observational cohort study will comprise at least 2,000 infants born across four districts of Uttar Pradesh, India. Community health workers, certified for GMA, video record and assess the infants' GMs twice, i.e. within 2 months after birth and at 3-5 months. In case of abnormal GMs and/or reduced motor optimality scores, infants are further examined by a paediatrician and a neurologist. If necessary, early intervention strategies (treatment as usual) are introduced. After paediatric and neurodevelopmental assessments at 12-24 months, outcomes are categorised as normal or neurological/developmental disorders. Research objective 1: to relate the GMA to the outcome at 12-24 months. Research objective 2: to investigate the impact of predefined exposures. Research objective 3: to evaluate the inter-scorer agreement of GMA.

Ethics and dissemination: G.A.N.E.S.H. received ethics approval from the Indian Government Chief Medical Officers of Varanasi and Mirzapur and from the Ramakrishna Mission Home of Service in Varanasi. GMA is a worldwide used diagnostic tool, approved by the Ethics Committee of the Medical University of Graz, Austria (27-388 ex 14/15). Apart from peer-reviewed publications, we are planning to deploy G.A.N.E.S.H. in other vulnerable settings.

Strengths and limitations of this study

- G.A.N.E.S.H. includes the application of a non-intrusive, highly reliable assessment tool (the Prechtl General Movement Assessment, GMA) to assist early identification of a high

For peer review only

risk for cerebral palsy and other neurological and developmental disorders in a low-income geographical cohort.

- G.A.N.E.S.H. aims to provide evidence as to whether a community-based identification and intervention programme facilitates early detection of neurological and developmental disorders and disabilities.
- Measurement biases may occur because blinding is not possible: it has taken the medical doctors and other staff members involved 15 years to win the vulnerable population's trust, which is the basis for conducting a study as outlined here.
- Higher-order impacts may be underestimated as a follow-up period of 24 months may be too short because certain neurological and developmental disorders cannot be diagnosed with absolute certainty at this age.
- Developmental delay in particular, may occur after weaning from breastfeeding because of undernourishment, and might therefore not be detected by means of GMA during the first months of life.

For peer review only

INTRODUCTION

Background and problem context

India has the world's largest birth ratio (about 26 million per year) and is experiencing dramatic improvements in infant and child survival, although neonatal and infant mortality rates are still high at 30 and 41 per 1,000 live births, respectively.[1] With a population of more than 199 million (ca. 16% of the national population), Uttar Pradesh is the most populous state in India. The 2011 census[2] showed that 59 million people in Uttar Pradesh live below the poverty line. The state's maternal mortality rate is the second highest in India with 285 maternal deaths per 100,000 live births. The under-five mortality rate is 90 per 1,000 live births.[3] Lack of antenatal care, unsafe child births, undernourishment, and lack of access to health care infrastructure are among the main risk factors for maternal and child morbidity and mortality. Neurological and developmental disorders and disabilities in particular compromise the attainment of full social and economic potential at family and community levels. The rate of occurrence of neurodevelopmental disorders ranges from 2.9% to 18.7% in 2- to 9-year-old children across five Indian regions, and its all-site pooled estimate is 9.2% for children younger than 6 years.[4] In other words, almost one in eleven children has at least one of the following impairments: neuromuscular disorders and cerebral palsy, epilepsy, intellectual disability, speech and language disorders, hearing and/or vision impairment, or autism.[4] Pooled estimates are unaffected by gender or rural/urban residence but increase by up to 3 percentage points when adjusted for national rates of low birth weight and/or stunting.[4] The overall pooled prevalence of cerebral palsy per 1,000 children in India is 2.95 (95% CI 2.03 to 3.88).[5]

Apart from low birth weight (<2500g) and preterm birth (< 37 weeks' gestation), non-institutional delivery, a history of perinatal asphyxia (delayed cry and difficult breathing at birth), neonatal illness requiring hospitalisation, postnatal brain infections, and stunting are significantly associated with neurological and developmental disorders and disabilities.[4,5] Several of these risk factors could be averted through public health intervention. There is strong evidence that early intervention improves functional outcomes for infants with neurological and developmental disorders and is economically cost-effective as it reduces the rate and severity of later impairments.[6,7] Early identification of neurodevelopmental impairment is therefore vital for ensuring timely referral to the limited resources provided for impoverished children. However,

For peer review only - http://bmjopen.bmj.com/site/about/guidelines.xhtml

low-income communities, which are most vulnerable to neurological and developmental disorders, often lack programmes for early identification and intervention.[8]

Government programmes such as India's Integrated Child Developmental Services provide supplementary food, health and nutrition education, health check-ups, growth monitoring and child-specific services like immunisation. But to this day only 20% to 30% of people from marginalised groups access these services.[9] Non-governmental organisations, and especially their community health workers, are indispensable for improving the access to early identification and intervention programmes since they work hand in hand with Accredited Social Health Activists (ASHAs), Auxiliary Nurse Midwives (ANMs) and Anganwadi Workers (AWWs), all of whom are part of India's public health care system. It is therefore of utmost importance that community health workers acquire relevant knowledge and skills to be able to recognise early markers for neurological and developmental disorders and refer infants in need to paediatric and neurological examinations. This goal has been supported by the Bill & Melinda Gates Foundation with a Grand Challenges Explorations grant for the development of smartphone-based applications as assistive tools.[10]

About the organisations involved

The impact is assessed by two non-governmental organisations, Kiran Society (KS) and Ramakrishna Mission (RKM), in ten blocks across four districts in the south east of Uttar Pradesh, India.

Kiran Society (KS) is a non-profit organisation situated near Varanasi (also known as Benares) on the banks of the river Ganges in Uttar Pradesh. Since 1990, KS has sought to empower marginalised groups and children with disabilities through rehabilitation, education, vocational training, social integration, and advocacy (www.kiranvillage.org). KS offers institution- and community-based rehabilitation services providing neurological examinations and interventions, physio-, occupational-, speech/language- and psychotherapy as well as corrective orthopaedic aids and appliances. Based on the WHO's community-based rehabilitation matrix and on three schemes, KS' community-based rehabilitation unit currently provides services for 200,000 people in the districts of Varanasi and Mirzapur. One of these schemes is the **G**eneral Movement

For peer review only - http://bmjopen.bmj.com/site/about/guidelines.xhtml

Assessment in **N**eonates for **E**arly Identification and Intervention, **S**ocial Support and **H**ealth Awareness (https://kiranvillage.org/?page_id=3450): G.A.N.E.S.H. was launched in 2018.

Ramakrishna Mission (RKM) is a worldwide organisation founded in 1900. Its Varanasi branch, the RKM Home of Service, has a 230-bed hospital, nine indoor wards and 15 outpatient departments with various diagnostic units. Supported by the WHO, the European Commission, the British Medical Association and others, RKM Varanasi launched its Health Promotion Programme in 2000. Its main objectives are health education, primary health care services and training of community-based personnel in Varanasi and nearby districts. Its 15 actions and research projects include the following three, which are particularly relevant for G.A.N.E.S.H.: (a) child health care education (esp. multimedia presentations on feeding practices, hygiene and sanitation); (b) training community health workers and traditional birth attendants in promoting mother and child health; and (c) mobile mother and child health clinics along with family planning and immunisation interventions. Since 2009, RKM has provided antenatal care and gynaecological services performed by mid-level women health workers in rural areas around Varanasi.

Both organisations' community health workers use mobile tools to pursue a broad range of health education goals including subjects such as vaccination, nutrition, hygiene and sanitation, but most importantly, they provide regular personal care. In addition, both organisations assist the most socio-economically disadvantaged families (especially pregnant women and lactating mothers, infants and young children) in terms of food supplements containing vitamins, iron and calcium.

Project overview and aims

The present observational study is being carried out in a population-based cohort of underprivileged infants in four districts of Uttar Pradesh, India. We are assessing the motor repertoire of individuals aged 1 to 5 months and its association with (a) demographic, pre- and perinatal risk factors, and (b) the neurodevelopmental outcome in the second year of life. The study is conducted by the two non-governmental organisations portrayed above, which have long

For peer review only - http://bmjopen.bmj.com/site/about/guidelines.xhtml

worked together and with governmental organisations in the target area. Disadvantaged families know that KS and RKM provide assistance in case of need.

The staff involved in both organisations received basic and advanced training in the Prechtl General Movement Assessment (GMA)[11], a diagnostic tool for early assessment of the integrity of the young nervous system. Training was provided by a licensed tutor of the GM Trust (C.E.), who also supervises the GMA and data evaluation during the project. Infants at high-risk for neurological and developmental disorders and disabilities are further examined by a paediatrician at RKM (S.V.) and a neurologist at KS (M.T.), who have worked together closely for more than 15 years. Another paediatrician is volunteering to assess infants and children in paediatric camps. All three of them are GMA-trained. KS has a special Medical Fund to guarantee that infants in need receive further diagnostic examination such as EEG, X-ray, MRI, or even genetic testing. If indicated, early physiotherapy is provided by KS staff, while both KS and RKM's teams provide basic health education and counselling.

We expect G.A.N.E.S.H. to improve health outcomes in children living in the target area. The main aims of this 3-year project are to (a) train community health workers to contribute to the identification of infants at high-risk for neurological and developmental disorders early on; (b) monitor further assessments in those infants; and (c) initiate early and targeted intervention procedures (Table 1; Figure 1). Early identification is based on detailed assessments of the spontaneous motor behaviour[11] of all eligible infants born in eight Varanasi villages, 19 Mirzapur villages, one Sonbhadra cluster of villages and one Azamgarh cluster of villages. Research objective 1 is to relate the quantity and quality of movement and postural patterns – which will be determined by means of the Prechtl GMA[11] at 1 to 5 months – to the outcome at 12 to 24 months. Outcome will be assessed by paediatric and neurological examination (including the Hammersmith Infant Neurological Examination[12]) as well as developmental testing (Trivandrum Developmental Screening Charts[13]). Research objective 2 is to investigate whether a number of predefined exposures such as (a) the access to and frequency of antenatal care, (b) maternal micronutrient deficit (such as iodine, iron and vitamin A deficiency) during pregnancy according to the medical history in case of institutional delivery, (c) safety of child birth (home vs. institutional delivery), (d) low birth weight (< 2500g) and undernourishment of the infant

For peer review only - http://bmjopen.bmj.com/site/about/guidelines.xhtml

For peer review only

(weight, length and head circumference at the time of GMA being 2*SD* below the reference group), and (e) intra-partum related complications (difficulties to breathe and/or delayed cry at birth) are associated with movements and postures assessed at 1 to 5 months. Research objective 3 is to examine inter-scorer agreement between community health workers and GMA experts.

METHODS AND ANALYSES

Study design

Observational cohort study which follows the Strengthening the Reporting of Observational Studies in Epidemiology (STROBE) guidelines.

Study setting and profile

In 2019, G.A.N.E.S.H. was implemented in 29 (cluster of) villages in ten blocks of four districts of Uttar Pradesh, where the burden of poverty and undernutrition is particularly high: the Varanasi district (roughly 3,700,000 inhabitants; 1,535 km^2; eight villages in the Kashi Vidyapeeth block, which is covered by KS); the Mirzapur district (roughly 2,500,000 inhabitants; 4,521 km^2; twelve villages in the Majhawan and Shikhar blocks, likewise covered by KS; seven villages or cluster of villages in the Chhanbey, Haliya, Jamalpur, Lalganj, Majhawan, Pahari and Patehara blocks, covered by RKM); the Sonbhadra district (roughly 1,800,000 inhabitants; 6,788 km^2; one cluster of villages in the Robertsganj block, covered by RKM), and the Azamgarh district (roughly 4,600.000 inhabitants; 4,054 km^2; one cluster of villages in the Lalganj block, covered by RKM).

Eastern Uttar Pradesh (particularly the districts of Mirzapur and Sonbhadra) has one of the lowest health indicators of India's eight socioeconomically backward states, also referred to as Empowered Action Group (EAG) states. Poor health is characterised by lack of nutrition, chronic diseases and a high prevalence of infectious diseases resulting not only in high mortality and morbidity but also in an increase of poverty due to out-of-pocket expenses and lost earnings.[14] The main sources of livelihood are agriculture, carpet weaving and pottery, but youth unemployment is high. According to the latest census from 2011[2], the literacy rate then was between 64% in the Sonbhadra district and 75% in the Varanasi district, with a 15 to 20% lower literacy rate in women.

For peer review only - http://bmjopen.bmj.com/site/about/guidelines.xhtml

The target area is characterised by a lack of trained medical staff willing to work in rural areas and a lack of health care infrastructure. Most of the care is provided by the family. Families who seek treatment will often turn to non-allopathic providers such as practitioners of Indian traditional medicine and quacks (i.e. unqualified and unskilled health practitioners pretending to have medical skills).[14] The lack of neurodevelopmental health professionals, particularly in rural areas, places specialist neurodevelopmental care out of the reach of most people. The G.A.N.E.S.H. programme addresses this critical issue and seeks to provide healthcare to families in need.

The target population of G.A.N.E.S.H. comprises approximately 50,000 people. We aim to assess a cohort of at least 2,000 infants born in the first and second years of the study (2019-2020) and collect their outcome data during the second and third years of the study (2020-2021). Due to the COVID-19 pandemic, data collection is deferred since March 15, 2020 and the timeline of the study will be re-scheduled.

In order to discuss the procedure and aims of the G.A.N.E.S.H programme, community health workers of KS and RKM arranged meetings with ASHAs and ANMs in each block. ANMs are village-based female health workers establishing the first contact between the community and health services; in remote areas, ANMs also conduct home deliveries;[15] usually each ANM is supported by four to five ASHAs. ASHAs are local women trained as health educators and appointed by the Ministry of Health and Family Welfare of India. Their tasks include trying to persuade women to give birth in hospitals, taking children to immunisation clinics, encouraging family planning, treating basic illnesses and injuries with first aid, and improving village sanitation.[15]

Each community health worker involved in the project is linked with one ANM and will be informed by them about each live birth in her village. Every Wednesday and Saturday, the government offers free services in primary health centres in our target areas, where ANMs and ASHAs check the infants' weight and growth, promote and assist with breastfeeding and other nutritional aspects, and vaccinate. Community health workers linked to G.A.N.E.S.H. can also recruit mothers with newborn and young infants there. The community health worker approaches

For peer review only - http://bmjopen.bmj.com/site/about/guidelines.xhtml

the parent(s) within 4 weeks after delivery and explains the G.A.N.E.S.H. project in detail, including, for example, mutual commitments. He/she hands out leaflets with relevant information provided in words (Hindi) and pictures. Parents/caregivers are assured of confidentiality and anonymity in reports and publications generated as part of G.A.N.E.S.H. Participation is voluntary, and families are assured that refusing to participate will not affect their access to care. If parents agree to participate, their (written) consent is obtained. Illiterate parents are informed in the presence of a literate witness and consent with their finger-print.

For peer review only

Participants

The birth rate in the target area varies from 19.9 to 28.7 per 1,000.[2] With a population of 50,000 we expect to see 1,000 to 1,500 newborns per year. Apart from living in the target area, inclusion criteria comprise all infants younger than completed 5 months of age irrespective of gender, family background, medical history and current health status. Infants older than 5 months of age are excluded.

Community health workers will collect demographic (please see also **Variables**), including the household composition and living condition, data from the mothers. Besides the medical history during pregnancy and delivery, maternal age and weight will also be documented.

The G.A.N.E.S.H. project is perceived by the communities as part of the ongoing services, which have been provided by KS and RKM for decades. More than 90% of newborn infants are brought to the free services in the primary health centers mentioned above, or, alternatively, the community health workers are welcomed at the family home. As part of the Indian culture, after having given birth, young mothers may move to the grandmother's home, possibly in another district. As the majority of the maternal grandparents also live in the same region, the number of moves to other regions is extremely small. Altogether, the number of families not willing to participate in the G.A.N.E.S.H. project is negligibly small.

Variables

After the recruitment of the infant, a community health worker collects the following data (i.e. exposure variables): demographical data (infant's gender, infant's age in days, parents' age,

For peer review only - http://bmjopen.bmj.com/site/about/guidelines.xhtml

marital status, number of children, family member with disability, living status and household composition, religious affiliation and caste); maternal medical condition, especially during pregnancy and delivery, according to the documentation of antenatal care and/or the medical history collected during institutional delivery; maternal nutritional status (weight and height); access to and frequency of antenatal care; site of delivery (home vs. institutional/hospital) indicating safety of child birth; complications during labour (documented in the medical history of the delivery) including intra-partum related complications such as difficulties to breathe and/or delayed cry at birth; the infant's gestational age at birth and birth weight. Frequency of antenatal care, age, weight, height, number of children, parental age and maternal weight are continuous data; all other data are categorical.

At 1 to 5 months we assess the following variables: (a) GMs: From birth to the end of the second month postterm age, we assess whether GMs are normal, poor repertoire, or cramped-synchronised[11] (categorical data). From the beginning of the third month to the end of the fifth month postterm age, we assess fidgety movements as normal, abnormal or absent[16] (categorical data) and (b) the motor optimality score[17] (MOS; ordinal data) and its five subscores (ordinal data) as well as the number of normal and abnormal movement and postural patterns (continuous data); (c) clinical data include status of breastfeeding and the infant's medical condition since birth such as fever, diarrhea (categorical data); (d) weight (measured with a portable weighing scale), length (measured on an infantometer), and head circumference (measured with a tape measure extending from the middle of the forehead to the farthest part in the rear of the head) are given as Z-scores, weight-for-age Z-scores, and weight-for-length Z-scores (continuous data).

The outcome at 12 to 24 months is either normal or classified as neurological and developmental disorders including cerebral palsy, epilepsy, (orthopaedic) congenital malformations, muscular dystrophy and spinal muscular atrophy, developmental delay, genetic disorders, visual impairment, hearing impairment, or a high risk for autism spectrum disorder (without the list being exhaustive; categorical data). The scores of the Hammersmith Infant Neurological Examination (HINE)[12] are ordinal data. In addition, each child's nutritional status is assessed and categorised as normal, underweight, wasted (weight-for-age Z-scores that are more than 2 standard deviations below the median of the WHO child growth reference standards), or stunted

For peer review only - http://bmjopen.bmj.com/site/about/guidelines.xhtml

(with height-for-age Z-scores that are more than 2 standard deviations below the median of the WHO child growth reference standards). Head circumference Z-scores (continuous data) will also be documented.

Data sources and measurement

All variables described in the first paragraph of the section **Variables** are obtained by a community health worker abiding by a predefined interaction protocol and, if available, from the medical records of delivery.

Motor behaviour at 1 to 5 months is assessed by means of the Prechtl General Movement Assessment (GMA),[11] which is widely regarded as the best clinical tool to predict cerebral palsy in infants under 5 months[18] and has proved invaluable for contributing to identify children at risk for developmental delay,[11] cognitive dysfunction,[19] intellectual disabilities,[20,21] speech and language disorders,[22] and autism spectrum disorders[23]. GMA is a diagnostic tool for an early assessment of the integrity of the young nervous system based on observation of young infants' spontaneous movements. It is entirely non-intrusive and extremely time-and cost-efficient. For a reliable assessment (kappa values ranging from 0.88 to 0.92)[11,17], a 2- to 5-minute video of the infant is recorded following a number of basic principles (infant lying in supine position, comfortably dressed, not crying, etc.).[11] Performed by trained assessors, the summary estimates for sensitivity and specificity of GMA are 0.98 (95% CI: 0.74–1.00) and 0.91 (95% CI: 0.83–0.93), respectively.[24] GMA is ideal for the neurological assessment of young infants, especially in low- and middle-income countries that struggle to provide expensive medical equipment for diagnoses.[25] From birth to the end of the second month postterm age, normal general movements (GMs) include a fluctuating sequence of arm, neck, trunk and leg movements. They wax and wane with changing intensity, speed and range of motion. Their onset and ending is typically gradual and smooth. Changing of direction and rotations across the trunk and limb axes are fluent and elegant.[11] From 3 to 5 months, GMs appear as fidgety movements with a very small amplitude and moderate speed of shoulders, wrists, hips and ankles in all directions and of variable acceleration.[11] Most infants will develop normally if fidgety movements are present and normal, especially if they co-occur with other smooth and fluent age-specific movement patterns.[11,16,19] The neural mechanisms of GMs are specific neural networks, central pattern

For peer review only

generators (CPG), which are most probably located in the brain stem. We believe that the CPGs for GMs are constantly modulated by more rostral parts of the brain. The resulting variable movements in turn modulate the CPG-activity by variable sensory feedback.[11,19] If a structural impairment of the young nervous system becomes functional, supra-spinal projections are impaired and reduce the modulation of CPG activity, resulting in less variable GMs.[19] Before 3 months of age, abnormal GMs are described as: (i) 'poor repertoire' (the sequence of movements and the intensity, speed and range of motion lack variability) or (ii) 'cramped-synchronised' (the limb and trunk muscles appear rigid and contract almost simultaneously, then relax almost simultaneously).[11] As a sign of functional impairment, fidgety movements can be (iii) 'abnormal' (exaggerated in amplitude and speed), (iv) sporadically present (not exceeding 3 seconds[26]) or (v) missing altogether at a postterm age of 9 to 16 weeks; (iv) and (v) are summarised as 'absent fidgety movements'.[11,16,26] Summary estimates of 0.98 for sensitivity and 0.91 for specificity make cramped-synchronised GMs followed by absent fidgety movements very reliable early markers of spastic cerebral palsy.[16-18,24]

The motor repertoire of infants aged 3 to 5 months consists of fidgety movements as well as other movement patterns such as swiping, wiggling-oscillating limb movements, kicking, arm and leg movements to the midline, fiddling, leg lifts, or arching.[17,27]The detailed GMA for 3- to 5-month-old infants comprises the following five subcategories: (i) temporal organisation and quality of fidgety movements; (ii) quality of other observed movement patterns; (iii) age-adequate movement repertoire; (iv) postural patterns; and (v) movement character. Adding the scores of the five subcategories reveals the motor optimality score (MOS)[11,17] with a maximum of 28 (i.e. best possible performance) and a minimum of 5. An MOS ranging from 25 to 28 is considered to be optimal; scores from 20 to 24 are mildly reduced; and an MOS below 20 requires intervention. A score below 9 indicates a very high risk for neurodevelopmental disabilities, especially for non-ambulatory cerebral palsy.[17] Various studies revealed that a reduced MOS was associated with later motor,[17,28-30] cognitive,[29,31] and language[22,29] impairments. A recent worldwide study on 468 children later diagnosed with cerebral palsy shows that such a detailed assessment of early movements and postures provides insight into a child's later functional capabilities and impairment.[17] Intra-observer reliability is high with intra-class correlation coefficients ranging from 0.80 to 0.98[17,28] and Cohen Kappa ranging from 0.87 to 0.91[30].

For peer review only - http://bmjopen.bmj.com/site/about/guidelines.xhtml

In February and November 2018, KS and RKM staff involved in G.A.N.E.S.H. (medical doctors, physiotherapists, medical social workers, community-based rehabilitation field workers, special educators, and one psychologist) received training from C.E.. They are now GM Trust-certified (basic and advanced level; www.general-movements-trust.info) to perform GMA. As a side note: for better readability we have summarised the professions of medical social workers, community-based rehabilitation field workers and special educators under the term ‘community health workers’ throughout the manuscript.

After parental consent has been received, a GMA-trained KS/RKM staff member videotapes the infant’s GMs according to GMA standards (the infant shows 2 to 5 minutes of active wakefulness, must not be crying, fussy or sucking on a pacifier, and must be lying in supine position without manipulation).[11] Video-recordings are preferably at two points in time: (a) during the first 2 months of life; and (b) during the fidgety movement period between the end of the 2nd month and the end of the 5th month postterm age (corrected for preterm delivery). Infants are videotaped in the parents’ home, in health camps, or in a primary health centre. The initial evaluation is performed by the GMA-certified staff member who has recorded the video, the second evaluation is performed by the first author (M.T.), and a third evaluation by C.E., who isn’t familiar with the medical history of the infant. C.E. supervises the GMA as licensed tutor of the GM Trust. In a pilot sampling approach, we recorded and assessed 400 infants revealing intra-class correlation coefficients ranging from 0.85 to 0.92 between the primary and secondary assessors and the supervisor.

The neurological assessment at 12 to 24 months follows the protocol of the HINE.[12] This method is easily performed and was designed for examining children between 2 and 24 months of age. It includes 26 items that assess cranial nerve function, posture, quality and quantity of movements, muscle tone, and reflexes and reactions. Each item is scored from 0 (minimum) to 3 (maximum score) revealing an overall global score from 0 to 78 (best neurological performance).[12] Both the sensitivity and the specificity are around 0.90.[32]

For peer review only - http://bmjopen.bmj.com/site/about/guidelines.xhtml

The Trivandrum Developmental Screening Chart (TDSC)[13] is based on 17 test items from the Bayley Scales of Infant Development (Baroda Norms). It was validated both at hospital and community levels and has a sensitivity of 0.85 and a specificity of 0.91, which makes it a simple, reliable screening tool that can be applied by community health workers.[33] The test-giver draws a vertical line through the chart indicating the actual age of the child. If the child can complete items that are to the left of the line, then there is no delay for that item. If an item lies to the left of the line and the child cannot complete this item, then an item delay is assumed. In this case, a trained assessor at KS will administrate the Bayley Scales of Infant and Toddler Development, Third Edition (cognition, language, motor, social-emotional, and adaptive behaviour domains) with the child. Bayley-III scores exceeding 85 are considered to be normal. In addition, two psychologists (one at KS, one at RKM) were trained to identify socio-communicative deficits which, along with repetitive and restricted behaviours, may indicate an increased likelihood of developing autism spectrum disorder.

A potential confounder especially after weaning from breastfeeding (usually after 6 months of age) might be undernutrition. It can be directly attributed to inadequate dietary intake, infection or disease that may affect the child. Lack of sanitation and hygiene, inadequate care, maternal mental health, economic deprivation, and food insecurity might also be factors influencing the child's development. Undernutrition and other external disadvantages hampering thriving will be revealed and documented at all assessment appointments.

Intervention programmes

The infant is referred to a targeted intervention programme if at least one of the following criteria is observed: (a) cramped-synchronised GMs during the first two months; in case of abnormal poor repertoire GMs during the first two months, GMA is repeated at 3 to 5 months, which will determine the further procedure; (b) absence of fidgety movements or exaggerated, abnormal fidgety movements at 3 to 5 months; (c) MOS <20; (d) asymmetries of any kind, especially asymmetric segmental wrist and/or finger movements[17]; (d) body weight and/or head circumference at or below the third age-related percentile on the WHO charts; (e) facial dysmorphism and/or malformations such as congenital talipes equinovarus, cleft palate; (f) acute illness; (g) any other parental concern.

For peer review only - http://bmjopen.bmj.com/site/about/guidelines.xhtml

Infants at high-risk for neurological and developmental disorders and disabilities and infants who need medical examination are referred to a paediatrician at RKM (S.V.) and/or a neurologist at KS (M.T.), who have worked together closely for more than 15 years. A second paediatrician is volunteering to assess infants and children in paediatric camps. All three of them are GMA-trained. KS has a special Medical Fund to guarantee that infants in need receive further diagnostic examination such as EEG, X-ray, MRI, or even genetic testing. If indicated, physio-, occupational-, speech/language therapists and special educators treat infants individually at home or at the Parents and Child Care Unit of KS. Since G.A.N.E.S.H. has been launched, every staff member involved in interventions has received regular workshops and practical demonstrations of early intervention techniques.

Trained RKM and KS personnel also offers education and counselling on basic health, nutrition and hygiene. Severely anaemic or malnourished mothers and underweight infants are given supplementary nutritious food, vitamins and micronutrients (especially calcium and vitamin D, iron and folic acid tablets) as well as medical care. Infants who need intensive medical treatment are referred to the Ramakrishna Hospital or the district hospitals of Varanasi and Mirzapur. Families in need are invited early on to attend paediatric or rehabilitation camps providing free services, examinations and, if necessary, additional medication.

Bias

Measurement biases may occur because blinding is not possible: it has taken the medical doctors and staff members 15 years to win the participating population’s trust, which is the basis for conducting this study. To avoid the interviewer bias, the community health worker will follow a predefined interaction protocol. In case the medical record of pregnancy and delivery is not available (lack of antenatal care or home delivery), a potential recall bias of the mother is hard to be ruled out. We will be able to control the confounders (e.g. undernutrition), as we will document any recognizable external disadvantages hampering thriving, and compare the Z-values of the anthropometric measurements at birth, 1 to 5 months, and 12 to 24 months.

Study size

For peer review only - http://bmjopen.bmj.com/site/about/guidelines.xhtml

Taken an alpha cut-off of 5% and a beta cut-off of 20%, the sample shall involve 1,261 children (i.e. with a pooled incidence of 9.2% neurodevelopmental disorders in Indian children younger than 6 years,[4] and an expected incidence of 7% in the study group, with a birth rate in the target area varying from 19.9 to 28.7 per 1,000[2], that is, we expect to see 1,000 to 1,500 newborns per year, resulting in about 70 to 105 children with neurological and developmental disorders and disabilities) to achieve a predictive power of .95 and an effect-size of .15 using Cohen's f^2. According to our eligibility criteria, we aimed to assess a cohort of at least 2,000 infants born in the first and second year of the study. Until March 15, 2020 (13 months after launching the study, before data collection was deferred due to the COVID-19 pandemic), we have successfully recruited 1,300 infants.

Statistical methods

Data will be analysed according to the research objectives of the study using IBM SPSS Statistics in its updated version with significance set at $p<0.05$. A lead biostatistician of the University Medical Center Goettingen, Germany, will provide expert input for the analyses.

Initial descriptive analysis will explore the characteristics and distributions of the variables. Any outlying data will be identified and the missing data explored. Histograms, boxplots and scatter plots, tables of means and SD or medians, interquartile ranges, and cross tabulations will be used as appropriate.

Sensitivity, specificity, and positive and negative predictive values will be determined with 95% CIs. This will be based on the positive / negative 'high risk of neurological and developmental disorders' status on GMs and MOS (at 3 to 5 months) compared with confirmed or suspected neurological and developmental disorders / no neurological and developmental disorders diagnoses (at 12 to 24 months). The association between the MOS (at 3 to 5 months) and the HINE (at 12 to 24 months) will be examined using Spearman's rank-order correlations. To test the predictive power of the MOS-subcategories (polytomous ordinal predictors) on the HINE cut-off outcomes (polytomous ordinal criterion), we will run ordinal regression analysis. The predictive power of abnormal movement and postural patterns (metric predictors) on HINE cut-

For peer review only - http://bmjopen.bmj.com/site/about/guidelines.xhtml

off outcomes (polytomous ordinal criterion) will be again estimated by means of ordinal regression analysis.

We will perform regression analyses to determine how exposure variables are related to GMs and MOS. General linear model analyses will be performed to understand the effect and interactions of predefined variables and covariates on GMs. In case of multiple subgroup comparisons, we will divide the significant level of 0.05 by the total number of subgroups of the analysis (Bonferroni correction).

GMs and MOS will be rated by at least one community health worker, the first (M.T.) and the third author (C.E.). Fleiss' kappa will be calculated to assess the agreement among the three scorers when assigning categorical ratings to GMs. Intra-class correlation coefficient (ICC) statistics will be applied to examine the agreement of the MOS among the scorers.

Longer-term outcomes

The listed longer-term outcomes are those we expect to achieve in a follow-up evaluation period of 3 to 5 years, depending on the availability of funds. Details will be determined later, as they can change according to requirements. Long-term outcomes should reflect an improved child health and nutritional status, improved motor, cognitive, and language abilities, a better supply of vision and hearing aids and orthoses, proper management in case of epilepsy, access to MRI, EEG, X-ray and other diagnostic procedures, as well as adequate psychological treatment and counselling – in other words: a proper management of neurological and developmental disorders and disabilities and a strengthened health system.

Patient and Public Involvement

It has not been appropriate to involve infants and their families or the public in the design, or conduct, or reporting, or dissemination plans of G.A.N.E.S.H.

Ethics and dissemination

G.A.N.E.S.H. received ethics approval from the Indian Government Chief Medical Officers of Varanasi and Mirzapur and from the Ramakrishna Mission Home of Service in Varanasi. GMA

For peer review only - http://bmjopen.bmj.com/site/about/guidelines.xhtml

is a worldwide used diagnostic tool, approved by the Ethics Committee of the Medical University of Graz, Austria (27-388 ex 14/15).

After parents/caregivers have received detailed information on G.A.N.E.S.H., they are assured of confidentiality and anonymity in reports and publications generated as part of G.A.N.E.S.H. Participation is voluntary, and families are assured that refusing to participate will not affect their access to care. If parents agree to participate, their (written) consent is obtained. Illiterate parents are informed in the presence of a literate witness and consent with their fingerprint. All study data are stored in secure databases. Paper files are kept in locked filing cabinets, and computer and video files are stored on password-protected servers. Only staff members associated with the G.A.N.E.S.H. programme have access to the data.

Results are submitted to peer-reviewed journals and presented at national and international conferences. Beyond that, we aim for G.A.N.E.S.H. to serve as a model for early identification of and intervention in children with (or a high-risk for) neurological and developmental disorders and disabilities, to have a sustainably impact on health care plans for low-income settings. Therefore, dissemination to health care professionals and policy makers is crucial.

All results presented are group data; individual participants are not identifiable. We intend to analyse and publish GMA and MOS results as well as 12-to 24-month outcomes as stand-alone research articles in peer-reviewed journals. In accordance with our main aim to improve the quality and quantity of interactions with beneficiary families, the main research questions (in chronological order) to answer and discuss, are:

1. Is it feasible for trained community health workers to launch G.A.N.E.S.H. and apply GMA in a rural, low-income setting? Key aspects: s*uccess of training, acceptance by ASHAs and ANMs, acceptance in the community, challenges, inter-scorer agreement, frequent staff meetings, expert supervision.* Limitations/risks: *parental refusal, cultural setting in case of male community health worker, migration of trained staff, priority of nutritional aspects over GMA.*
2. Does G.A.N.E.S.H. improve the frequency of home visits for infants and their mothers and the extent and level of counselling for families in need, and does it help to identify

For peer review only - http://bmjopen.bmj.com/site/about/guidelines.xhtml

abnormal motor behaviour and malnutrition? Key aspects: *identification of abnormal GMs and reduced MOS, parental compliance with further paediatric and neurological examination, launching of targeted family-oriented intervention, targeted health education, provision of food supplementation and micronutrients.* Limitations/risks: *no compliance with further assessment and intervention because early markers are subtle; families see the need to consult a doctor only in case of (frequent) seizures, acute infection, or malformations like cleft palate and congenital talipes equinovarus. The need for prevention and early intervention is difficult to grasp for parents, and is often impeded by cultural hierarchical family structure.*

3. Are we able to identify early on (especially at 3 to 5 months) children who will have an adverse outcome around 12 to 24 months of age? Key aspects: *predictive value of GMA, sustainable family acceptance and compliance with targeted strategies of early intervention.* Limitations/risks: *the nutritional status, especially after weaning from breastfeeding (second half of the first year of life) might have a greater impact on the development than the integrity of the nervous system determined by GMA.*

Acknowledgements: The authors are grateful to Professor Akmer Mutlu, Hacettepe University Ankara, Turkey for providing teaching material to the physiotherapy unit at Kiran Society; to Mrs. Sikha Sharma for basic insights into the principles of early intervention; to Mr. Amarendra Kumar and Mr. Vijay Pal for their support in community-based rehabilitation; to Dr Ruchi Pandey for her assistance in paediatric camps; to Mrs. Sangeeta J.K., founder and president of Kiran Society, for introducing the community-based rehabilitation approach among Kiran Society's activities and her ongoing support and inspiration; and to Miha Tavcar (www.scriptophil.at) for copy-editing the article.

Contributors statement:

Moreno Toldo conceptualised G.A.N.E.S.H. and conceived the research questions; he is principle investigator and secured funding for the project; he prepared the study protocol with feedback from all collaborators; he provides second opinions for GMA and

For peer review only - http://bmjopen.bmj.com/site/about/guidelines.xhtml

supervises and provides neurological interventions; he performs HINE and trained the physiotherapists in the same; he drafted the manuscript and approved the final manuscript as submitted.

Swami Varishthananda conceptualised G.A.N.E.S.H. and conceived the research questions; he is principle investigator and secured funding for the project; he prepared the study protocol with feedback from all collaborators; he supervised and provided paediatric interventions applied in G.A.N.E.S.H., he provided critical review of the first manuscript and the first revision.

Christa Einspieler conceptualised G.A.N.E.S.H. and conceived the research questions; she is principle investigator and secured funding for the project; she prepared the study protocol with feedback from all collaborators; she developed the data base for the project; she trained all staff members in GMA, supervises GMA and provides third opinions. She drafted the manuscript, its revisions and approved the final manuscript as submitted.

Neeraj Tripathi supervises the data collection under the guidance of investigators; he developed the data base for the project; he supervises and provides physiotherapeutic interventions; he performs HINE and approved the final manuscript as submitted.

Anshu Singh supervises the data collection under the guidance of investigators; she developed the data base for the project; she supervises and provides health education including antenatal care; she performs GMA, provided critical review of the manuscript and approved the final manuscript as submitted.

Surendra K. Verma developed the data base for the project and performs GMA and TDSC; he approved the final manuscript as submitted.

Kanchan Vishwakarma supervises the data collection under the guidance of investigators; she performs GMA and provides health education including antenatal care; she approved the final manuscript as submitted.

Dajie Zhang advised on statistical design of the study, provided critical review of the first and second revisions and approved the final manuscript as submitted.

Agyeya Dwivedi provides physiotherapeutic intervention and health education; he performs HINE and TDSC; he approved the final manuscript as submitted.

Ritika Gupta performs GMA and provides health education; she approved the final manuscript as submitted.

For peer review only

Sanjay Karn performs GMA and provides health education, he approved the final manuscript as submitted.

Nirmal Kerketta provides physiotherapeutic intervention and health education, he performs HINE and TDSC, and approved the final manuscript as submitted.

Ram Narayan performs GMA and TDSC, and provides health education. He approved the final manuscript as submitted.

Karuna Nikam Singh provided expert advice on early intervention in low-resource settings and supervises interventions applied in G.A.N.E.S.H.; she approved the final manuscript as submitted.

Sumitra Rani performs GMA and provides health education and approved the final manuscript as submitted.

Akanksha Singh developed the data base for the project; she performs GMA and provides health education including antenatal care; she approved the final manuscript as submitted.

Divyanshu Singh provides physiotherapeutic intervention and health education; he performs HINE and TDSC. He approved the final manuscript as submitted.

Krishna P. Singh performs GMA and provides health education; he approved the final manuscript as submitted.

Navin Singh provides hearing assessments and coordinates physiotherapeutic interventions. He approved the final manuscript as submitted.

Neeraj Singh provides physiotherapeutic intervention and health education; he performs HINE and TDSC, and approved the final manuscript as submitted.

Rishi Singh performs GMA and provides health education; she approved the final manuscript as submitted.

Shyam P. Singh performs GMA and provides health education; he approved the final manuscript as submitted.

Rakesh Srivastava he supervises and provides physiotherapeutic interventions and performs HINE; he approved the final manuscript as submitted.

Sandeep Srivastava performs GMA and TDSC; he provides special education and approved the final manuscript as submitted.

Sanjeev Srivastava performs GMA and provides health education; he approved the final manuscript as submitted.

For peer review only - http://bmjopen.bmj.com/site/about/guidelines.xhtml

Gopal Yadav performs GMA, HINE and TDSC; he approved the final manuscript as submitted.

Preeti Yadav performs GMA and TDSC and provides health education; she approved the final manuscript as submitted.

Sheshnath Yadav performs GMA and provides health education; she approved the final manuscript as submitted.

Sujata Yadav performs GMA and provides health education; she approved the final manuscript as submitted.

Peter B. Marschik is co-PI; he secured funding for the project, assisted in conceptualisation and prepared the study protocol with feedback from all collaborators; he provided critical review of the manuscript and approved the final manuscript as submitted.

Funding: This work is supported by Bill & Melinda Gates Foundation (opp1128871), the Austrian Science Fund FWF (P25241, TCS24, KLI811), the GM Trust (WHEP23-8-5-16), the Australian Cerebral Palsy Alliance (SEG00419), the Old Possum's Practical Trust, the Medical Fund of Kiran Society and the Ramakrishna Mission.

Competing interests: The GM Trust supports G.A.N.E.S.H.. CE is a GM Trust tutor for GMA, and PBM is president of the GM Trust (honorary function), both of whom have contributed to G.A.N.E.S.H. on an honorary basis.

Ethics Approval: G.A.N.E.S.H. received ethics approval from the Indian Government Chief Medical Officers of Varanasi and Mirzapur and from the Ramakrishna Mission Home of Service in Varanasi. GMA is a worldwide used diagnostic tool, approved for its retrospective and prospective application by the Ethics Committee of the Medical University of Graz, Austria (27-388 ex 14/15).

Provenance and peer review: Not commissioned; externally peer-reviewed.

Data sharing statement: This being a protocol no unpublished data are available.

REFERENCES

For peer review only - http://bmjopen.bmj.com/site/about/guidelines.xhtml

1. International Institute for Population Sciences (IIPS) and ICF. National Family Health Survey (NFHS-4), 2015-2016: India. Mumbai: IIPS, 2017. http://rchiips.org/NFHS/NFHS-4Reports/India.pdf (accessed Nov 25, 2019).
2. Census of India 2011. www.censusindia.gov.in (accessed Oct 3, 2019).
3. Annual Health Survey Report, Part I. Office of the Registrar General & Census Commissioner. Ministry of Home Affairs, Government of India, 2013.
4. Arora NK, Nair MKC, Gulati S, *et al*. Neurodevelopmental disorders in children aged 2–9 years: Population-based burden estimates across five regions in India. *PLoS Med* 2018;15:e1002615.
5. Chauhan A, Singh M, Jaiswal N, *et al*. Prevalence of cerebral palsy in Indian children: a systematic review and meta-analysis. *Indian J Pediatr* 2019;doi 10.1007/s12098-019-03024-0.
6. Spittle A, Orton J, Anderson P, *et al*. Early developmental intervention programmes post-hospital discharge to prevent motor and cognitive impairments in preterm infants. *Cochrane Database Syst Rev* 2015;11:CD005495.
7. Benfer KA, Novak I, Morgan C, *et al*. Community-based parent-delivered early detection and intervention programme for infants at high risk of cerebral palsy in a low-resource country (Learning through Everyday Activities with Parents (LEAP-CP): protocol for a randomized controlled trial. *BMJ Open* 2018;8:e021186.
8. UN World Health Organization (WHO). 58th World Health Assembly: Resolutions and Decisions, Annex. Geneva: World Health Organization 2005. Document WHA58/2005/REC/1. http://apps.who.int/gb/ebwha/pdf_files/WHA58-REC1/english/A58_2005_REC1-en.pdf (accessed at July 15, 2019).
9. Chakrabarti S, Raghunathan K, Alderman H, *et al*. India's Integrated Child Development Services programme; equity and extent of coverage in 2006 and 2016. *Bull World Health Organ* 2019;97:270-282.
10. Marschik PB, Pokorny FB, Peharz R, *et al*. A novel way to measure and predict development: a heuristic approach to facilitate the early detection of neurodevelopmental disorders. *Curr Neurol Neurosci Rep* 2017;17:43. doi 10.1007/s11910-017-0748-8

For peer review only - http://bmjopen.bmj.com/site/about/guidelines.xhtml

11. Einspieler C, Prechtl HFR, Bos AF, *et al. Prechtl's Method on the Qualitative Assessment of General Movements in Preterm, Term and Young Infants*. London, UK: Mac Keith Press 2004.

12. Haataja L, Mercuri E, Regev R, *et al*. Optimality score for the neurologic examination of the infant at 12 and 18 months of age. *J Pediatr* 1999;135:153-161.

13. Nair MK, George B, Philip E, *et al*. Trivandrum developmental screening chart. *Indian Pediatr* 1991;28:869-872.

14. Arokiasamy P, Gautam A. Neonatal mortality in the empowered action group states of India: trends and determinants. *J Biosoc Sci* 2008;40:183-201.

15. Nimmagadda S, Gopalakrishnan L, Avula R, *et al*. Effects of an mHealth intervention for community health workers on maternal and child nutrition and health services delivery in India: protocol for a quasi-experimental mixed-methods evaluation. *BMJ Open* 2019;9:e025774.

16. Prechtl HFR, Einspieler C, Cioni G, *et al.* An early marker for neurological deficits after perinatal brain lesions. *Lancet* 1997;349:1361-1363.

17. Einspieler C, Bos AF, Krieber-Tomantschger M, *et al*. Cerebral palsy: early markers of clinical phenotype and functional outcome. *J Clin Med* 2019;8:1616. doi: 10.3390/jcm8101616.

18. Novak I, Morgan C, Adde, L, *et al.* Early, accurate diagnosis and early intervention in cerebral palsy: advances in diagnosis and treatment. *JAMA Pediatr* 2017;171:897-907.

19. Einspieler C, Bos AF, Libertus ME, *et al*. The general movement assessment helps us to identify preterm infants at risk for cognitive dysfunction. *Front Psychol* 2016;7:406. doi 10.3389/fpsyg.2016.00406.

20. Herrero D, Einspieler C, Panvequio Aizawa CY, *et al*. The motor repertoire in 3- to 5-month old infants with Down syndrome. *Res Dev Disabil* 2017;67:1-8.

21. Einspieler C, Marschik PB. Regression in Rett syndrome: Developmental pathways to its onset. *Neurosci Biobehav Rev* 2019,98:320-332.

22. Salavati S, Einspieler C, Vagelli G, *et al*. The association between the early motor repertoire and language development in term children born after normal pregnancy. *Early Hum Dev* 2017;111:30-35.

For peer review only - http://bmjopen.bmj.com/site/about/guidelines.xhtml

23. Einspieler C, Sigafoos J, Bartl-Pokorny KD, *et al.* Highlighting the first 5 months of life: general movements in infants later diagnosed with autism spectrum disorder or Rett syndrome. *Res Autism Spectr Dis* 2014;8:286-291.

24. Bosanquet M, Copeland L, Ware R, *et al.* A systematic review of tests to predict cerebral palsy in young children. *Dev Med Child Neurol* 2013;55:418-426.

25. Tomantschger I, Herrero D, Einspieler C, *et al.* The general movement assessment in non-European low- and middle-income countries. *Rev Saude Publica* 2018;52:6. doi: 10.11606/S1518-8787.2018052000332.

26. Einspieler C, Yang H, Bartl-Pokorny KD, *et al.* Are sporadic fidgety movements as clinically relevant as is their absence? *Early Hum Dev* 2015;91:247-252.

27. Einspieler C, Marschik PB, Prechtl HFR. Human motor behaviour. Prenatal origin and early postnatal development. *J Psychol* 2008;216:148-154.

28. Zang FF, Yang H, Han Q, *et al.* Very low birth weight infants in China: the predictive value of the motor repertoire at 3 to 5 months for the motor performance at 12 months. *Early Hum Dev* 2016;100:27-32.

29. Einspieler C, Utsch F, Brasil P, *et al.* Association of infants exposed to prenatal Zika virus infection with their clinical, neurologic, and developmental status evaluated via the general movement assessment tool. *JAMA Netw Open* 2019a; e187235.

30. Bruggink JL, Einspieler C, Butcher PR, *et al.* Quantitative aspects of the early motor repertoire in preterm infants: do they predict minor neurological dysfunction at school age? *Early Hum Dev* 2009;85:25-36.

31. Butcher PR, van Braeckel K, Bouma A, *et al.* The quality of preterm infants' spontaneous movements: an early indicator of intelligence and behaviour at school age. *J Child Psychol Psychiatry* 2009;50:920-30.

32. Romeo DM, Ricci D, Brogna C, *et al.* Use of the Hammersmith Infant Neurological Examination in infants with cerebral palsy: a critical review of the literature. *Dev Med Child Neurol* 2016;58:240-245.

33. Nair MK, Nair GS, George B, *et al.* Development and validation of Trivandrum Developmental Screening Chart for children aged 0-6 years [TDSC (0-6)]. *Indian J Pediatr* 2013;80:S248-S255.

For peer review only - http://bmjopen.bmj.com/site/about/guidelines.xhtml

Table 1: Main procedures and outcome measures

Procedures	Output	
Training community health workers to identify infants at high-risk for neurological and developmental disorders and disabilities early on	(1)	staff trained and certified in basic and advanced GMA
	(2)	agreement (a) between the scorers, (b) between the scorers and a GMA-trained neurologist, and (c) between the scorers and a GMA expert
Monitoring further assessments of infants at high-risk for neurological and developmental disorders and disabilities	(3)	number of infants screened
	(4)	number of infants identified (abnormal age-specific GMs, reduced MOS)
	(5)	number of families supported
	(6)	number of underweight infants
	(7)	number of infants examined by a paediatrician and/or a neurologist; examination results
	(8)	number of (breastfeeding) mothers and infants who received micronutrient supply
	(9)	number of infants who received (financially supported) access to MRI, EEG, X-ray, and laboratory testing
Launching targeted intervention procedures early on.	(10)	number of staff trained in early parent oriented intervention
	(11)	number of infants referred to early intervention
	(12)	number of families referred to further parent training (around the infant's age of 6 to 7 months)

Key: EEG, electroencephalography, GMA, general movement assessment; MOS, motor optimality score; MRI, magnetic resonance imaging

For peer review only - http://bmjopen.bmj.com/site/about/guidelines.xhtml

Legend to Figure 1:

Study design of the G.A.N.E.S.H. programme

Key: GMA, general movement assessment; MOS, motor optimality score; PTA, postterm age; TAU, treatment as usual

For peer review only

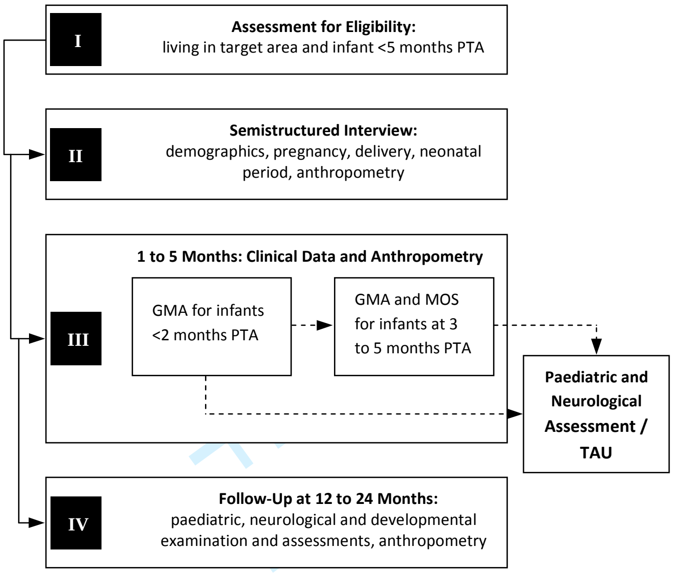

For peer review only

STROBE Statement—Checklist of items that should be included in reports of ***cohort studies***

	Item No	Recommendation	Page No
**Title and abstract**	1	(*a*) Indicate the study's design with a commonly used term in the title or the abstract	1
		(*b*) Provide in the abstract an informative and balanced summary of what was done and what was found	1
**Introduction**			
Background/rationale	2	Explain the scientific background and rationale for the investigation being reported	3-6
Objectives	3	State specific objectives, including any prespecified hypotheses	6-7
**Methods**			
Study design	4	Present key elements of study design early in the paper	7
Setting	5	Describe the setting, locations, and relevant dates, including periods of recruitment, exposure, follow-up, and data collection	7-9
Participants	6	(*a*) Give the eligibility criteria, and the sources and methods of selection of participants. Describe methods of follow-up	9
		(*b*) For matched studies, give matching criteria and number of exposed and unexposed	
Variables	7	Clearly define all outcomes, exposures, predictors, potential confounders, and effect modifiers. Give diagnostic criteria, if applicable	9-10
Data sources/ measurement	8*	For each variable of interest, give sources of data and details of methods of assessment (measurement). Describe comparability of assessment methods if there is more than one group	10-14
Bias	9	Describe any efforts to address potential sources of bias	15
Study size	10	Explain how the study size was arrived at	15-16
Quantitative variables	11	Explain how quantitative variables were handled in the analyses. If applicable, describe which groupings were chosen and why	9-10
Statistical methods	12	(*a*) Describe all statistical methods, including those used to control for confounding	16-17
		(*b*) Describe any methods used to examine subgroups and interactions	16
		(*c*) Explain how missing data were addressed	16
		(*d*) If applicable, explain how loss to follow-up was addressed	
		(*e*) Describe any sensitivity analyses	16
**Results**			
Participants	13*	(a) Report numbers of individuals at each stage of study—eg numbers potentially eligible, examined for eligibility, confirmed eligible, included in the study, completing follow-up, and analysed	n.a.
		(b) Give reasons for non-participation at each stage	
		(c) Consider use of a flow diagram	
Descriptive data	14*	(a) Give characteristics of study participants (eg demographic, clinical, social) and information on exposures and potential confounders	n.a.
		(b) Indicate number of participants with missing data for each variable of interest	
		(c) Summarise follow-up time (eg, average and total amount)	
Outcome data	15*	Report numbers of outcome events or summary measures over time	n.a.

For peer review only - http://bmjopen.bmj.com/site/about/guidelines.xhtml

Main results	16	(*a*) Give unadjusted estimates and, if applicable, confounder-adjusted estimates and their precision (eg, 95% confidence interval). Make clear which confounders were adjusted for and why they were included<br>(*b*) Report category boundaries when continuous variables were categorized<br>(*c*) If relevant, consider translating estimates of relative risk into absolute risk for a meaningful time period	n.a
Other analyses	17	Report other analyses done—eg analyses of subgroups and interactions, and sensitivity analyses	
**Discussion**			
Key results	18	Summarise key results with reference to study objectives	n.a.
Limitations	19	Discuss limitations of the study, taking into account sources of potential bias or imprecision. Discuss both direction and magnitude of any potential bias	
Interpretation	20	Give a cautious overall interpretation of results considering objectives, limitations, multiplicity of analyses, results from similar studies, and other relevant evidence	
Generalisability	21	Discuss the generalisability (external validity) of the study results	
**Other information**			
Funding	22	Give the source of funding and the role of the funders for the present study and, if applicable, for the original study on which the present article is based	19-20

*Give information separately for exposed and unexposed groups.

Note: An Explanation and Elaboration article discusses each checklist item and gives methodological background and published examples of transparent reporting. The STROBE checklist is best used in conjunction with this article (freely available on the Web sites of PLoS Medicine at http://www.plosmedicine.org/, Annals of Internal Medicine at http://www.annals.org/, and Epidemiology at http://www.epidem.com/). Information on the STROBE Initiative is available at http://www.strobe-statement.org.

For peer review only

For peer review only - http://bmjopen.bmj.com/site/about/guidelines.xhtml
